# Type 2 diabetes remission 1 year after an intensive lifestyle intervention: A secondary analysis of a randomized clinical trial

**DOI:** 10.1111/dom.13802

**Published:** 2019-06-30

**Authors:** Mathias Ried‐Larsen, Mette Y. Johansen, Christopher S. MacDonald, Katrine B. Hansen, Robin Christensen, Anne‐Sophie Wedell‐Neergaard, Nanna Skytt Pilmark, Henning Langberg, Allan A. Vaag, Bente K. Pedersen, Kristian Karstoft

**Affiliations:** ^1^ Centre of Inflammation and Metabolism and Centre for Physical Activity Research, Rigshospitalet University of Copenhagen Copenhagen Denmark; ^2^ CopenRehab, Section of Social Medicine, Department of Public Health, Faculty of Health and Medical Sciences University of Copenhagen Copenhagen Denmark; ^3^ Musculoskeletal Statistics Unit Parker Institute, Bispebjerg and Frederiksberg Hospital Copenhagen Denmark; ^4^ Research Unit of Rheumatology, Department of Clinical Research University of Southern Denmark, Odense University Hospital Odense Denmark; ^5^ Cardiovascular and Metabolic Disease Translational Medicine Unit, Early Clinical Development, IMED Biotech Unit AstraZeneca Gothenburg Sweden; ^6^ Department of Clinical Pharmacology Bispebjerg Hospital, University of Copenhagen Copenhagen Denmark

**Keywords:** clinical trial, dietary intervention, exercise intervention, type 2 diabetes, weight control

## Abstract

**Aim:**

To investigate whether an intensive lifestyle intervention induces partial or complete type 2 diabetes (T2D) remission.

**Materials and methods:**

In a secondary analysis of a randomized, assessor‐blinded, single‐centre trial, people with non‐insulin‐dependent T2D (duration <10 years), were randomly assigned (2:1, stratified by sex, from April 2015 to August 2016) to a lifestyle intervention group (n = 64) or a standard care group (n = 34). The primary outcome was partial or complete T2D remission, defined as non‐diabetic glycaemia with no glucose‐lowering medication at the outcome assessments at both 12 and 24 months from baseline. All participants received standard care, with standardized, blinded, target‐driven medical therapy during the initial 12 months. The lifestyle intervention included 5‐ to 6‐weekly aerobic and combined aerobic and strength training sessions (30‐60 minutes) and individual dietary plans aiming for body mass index ≤25 kg/m^2^. No intervention was provided during the 12‐month follow‐up period.

**Results:**

Of the 98 randomized participants, 93 completed follow‐up (mean [SD] age 54.6 [8.9] years; 46 women [43%], mean [SD] baseline glycated haemoglobin 49.3 [9.3] mmol/mol). At follow‐up, 23% of participants (n = 14) in the intervention and 7% (n = 2) in the standard care group met the criteria for any T2D remission (odds ratio [OR] 4.4, 95% confidence interval [CI] 0.8‐21.4]; *P* = 0.08). Assuming participants lost to follow‐up (n = 5) had relapsed, the OR for T2D remission was 4.4 (95% CI 1.0–19.8; *P* = 0.048).

**Conclusions:**

The statistically nonsignificant threefold increased remission rate of T2D in the lifestyle intervention group calls for further large‐scale studies to understand how to implement sustainable lifestyle interventions among people with T2D.

## 1 INTRODUCTION

Type 2 diabetes (T2D) is, in general, considered to be a progressive chronic condition with few prospects of reversal^1^; however, T2D may be reverted by Roux‐en‐Y gastric bypass, gastric sleeve surgery,^2^, ^3^, ^4^, ^5^ or through lifestyle interventions.^6^, ^7^, ^8^, ^9^ Recent studies have shown that the T2D remission rates using very‐low‐calorie diets are greater in people with T2D of short duration,^7^, ^8^, ^9^ with the potential to re‐establish pancreatic β‐cell function among the patients with the shortest disease duration.^10^ Studies on very‐low‐calorie diets, however, either did not increase physical activity levels among the participants or did not prioritize increasing physical activity.^8^, ^9^

Physical activity improves glycaemic control^11^ and may counteract inflammatory pathways related to pancreatic β‐cell dysfunction.^12^, ^13^, ^14^ Thus, it may be speculated that focusing on increased levels of exercise may facilitate T2D remission in parallel with a dietary intervention. In the Action for Health in Diabetes (Look AHEAD) trial, exercise was prioritized, but only a limited proportion of participants reached the levels of exercise prescribed.^15^ Furthermore, only modest T2D remission rates were seen in the Look AHEAD trial^6^; therefore, it may be possible that a lifestyle intervention implementing a higher exercise volume would increase T2D remission rates. In a previous study, we reported that >50% of participants undergoing an intensive lifestyle intervention with a focus on high volumes of exercise discontinued their glucose‐lowering pharmaceutical therapy, with concomitant improvements in glycated haemoglobin (HbA1c) levels after a 12‐month intervention indicating the potential for T2D remission.^16^ Nevertheless, the follow‐up time precluded inferences about the long‐term rate of T2D remission.^17^ Recently, we conducted a observational assessment 12 months after the termination of the intervention,^18^ allowing evaluation of T2D remission.

The aim of the present study was to test the hypothesis that an intensive exercise‐based lifestyle intervention, U‐TURN, is superior in introducing any T2D remission (partial or complete) compared to standard diabetes care 1 year after termination of the lifestyle intervention in participants with short‐lasting (<10 years) T2D.^16^

## 2 METHODS

### 2.1 Study design

The present extension study was designed as a pragmatic superiority study based on follow‐up assessment of the participants in a recent single‐centre clinical trial (Appendix S1),^18^ conducted in the Capital Region of Denmark from 29 April 2015 to 17 August 2017 and including 98 participants. The primary outcome was assessed after a 12‐month intervention and published previously.^16^ The data for the present extension study were based on the follow‐up examinations 12 months after the primary outcome assessment (ie, 24 months from the baseline assessments). Guidelines from the Helsinki Declaration were followed and reporting in this article is aligned with CONSORT standards. The study was approved by the Scientific Ethics Committee at the Capital Region of Denmark. All participants provided oral and written informed consent. The full trial protocol is provided in Appendix S1. The study was registered at ClinicalTrials.gov (NCT02417012).

### 2.2 Participants and eligibility

The original trial comprised 98 people with T2D, randomly allocated to either the intensive lifestyle intervention U‐TURN or standard care. Detailed recruitment, pre‐study procedures, randomization and allocation procedures are described in detail elsewhere.^11^, ^16^ No blinding was possible for this follow‐up investigation. The original eligibility criteria were T2D diagnosed within 10 years of entry to the study, receiving ≤2 glucose‐lowering medications and a body mass index (BMI) of 25 to 40 kg/m^2^. People who were insulin‐dependent, or had one or more of the following complications: diabetic retinopathy; macro‐albuminuria (urine albumin‐creatinine ratio ≥ 300 mg/g); nephropathy (plasma creatinine ≥1.47 mg/dL); or HbA1c level > 56 mmol/mol (9%) were not considered eligible.

The full analysis set for the present study was derived from the set of all randomized participants by minimal and justified elimination of participants^16^; participants allocated to a treatment group (U‐TURN or standard care) were followed up, assessed and analysed as members of that group, irrespective of their compliance to the treatment allocation^18^ (intention‐to‐treat population). All participants originally allocated to either the U‐TURN or the standard care group were invited to participate in the follow‐up assessments 24 months after the baseline assessment unless they explicitly declined further participation (n = 5); a total of 93 participants (95% of the original sample^16^) were invited to participate in the follow‐up procedures.

### 2.3 Interventions

Following the baseline procedures and until 12‐month follow‐up, all participants received standard care consisting of blinded, target‐driven pharmaceutical therapy (glucose‐, blood pressure‐ and lipid‐lowering) and lifestyle advice delivered by a diabetes nurse every third month. Specific therapeutic goals and procedures are described elsewhere.^18^ In addition, participants in the U‐TURN lifestyle intervention group received supervised resistance and aerobic exercise for 30 to 60 minutes, on 5 or 6 days per week. Participants randomized to the U‐TURN intervention were also provided with individually tailored dietary plans (macronutrient distribution: 45%‐60% carbohydrate, 15%‐20% protein, and 20%‐35% fat, with <7% saturated fat) with an energy intake restriction during the initial 4 months of the intervention.^18^ During the last 8 months of the intervention, the aim was to achieve energy balance. No interventions were provided after the assessments at 12‐month follow‐up.

### 2.4 Outcomes and procedures

The primary outcome for the present extension study was the prevalence of participants in each group with partial or complete remission of T2D when assessed after 24 months; this was defined according to the American Diabetes Association consensus statement as the presence of all four of the following criteria: (a) fasting glucose ≤6.9 mmol/L, (b) HbA1c < 48 mmol/mol (6.5%), (c) no glucose‐lowering medications at the outcome assessments, and (d) meeting these targets at both 12‐ and 24‐month follow‐up.^17^

Secondary outcome measures included changes from baseline to 24‐month follow‐up in HbA1c, 2‐hour glucose concentration after an oral glucose tolerance test, fasting glucose, fasting insulin, maximal oxygen uptake, weight, BMI, fat mass (total and abdominal), lean body mass, total cholesterol, LDL cholesterol, HDL cholesterol, triglycerides, systolic and diastolic blood pressure, energy intake and self‐reported physical activity energy expenditure. Between‐group differences at 24‐month follow‐up in the proportion of participants who obtained 5% or 10% weight loss, and complete T2D remission (analogous to partial remission but with fasting glucose <5.6 mmol/L and HbA1c <39 mmol/mol) were assessed,^17^, ^19^ as well as the proportion of participants with no need for glucose‐lowering medication (HbA1c ≤48 mmol/mol and no glucose‐lowering medication), no need for lipid‐lowering medication (LDL cholesterol <2.5 mmol/L and triglycerides ≤5.0 mmol/L with no lipid‐lowering medication), no need for blood pressure‐lowering medication (systolic blood pressure ≤ 130 mmHg and diastolic blood pressure ≤ 80 mmHg with no blood pressure‐lowering medication) at 24‐month follow‐up. Adverse events from baseline to 24‐month follow‐up were also reported.

Planned subgroup analyses assessing partial and complete T2D remission, were performed according to phenotypic subgroups: sex; age; T2D duration at entry to the trial and baseline risk factors; impaired glucose tolerance (glucose value in 2‐hour oral glucose tolerance test ≥11.1 mmol/L); impaired fasting glucose (fasting glucose ≥7.0 mmol/L); combination of impaired glucose tolerance and fasting glucose; low cardiorespiratory fitness^20^; and low need for glucose‐lowering medication (≤1000 mg metformin only). A subgroup analysis assessing remission was also performed per protocol from baseline to 12‐month follow‐up.^21^

All assessments were performed in one laboratory and the biochemical analyses were completed at the central laboratory (Rigshospitalet, Copenhagen, Denmark) using standard procedures as described previously.^18^, ^21^

### 2.5 Statistical analysis

The analysis of the primary outcome was performed according to the intention‐to‐treat principle using logistic regression analyses.^18^ Participants with outcome data indicating no, partial or complete T2D remission at 12‐month follow‐up were included in the analyses independent of participation in the 24‐month follow‐up assessment. When zero‐event data were observed, a continuity correction was employed that was inversely proportional to the relative size of the opposite group.^22^, ^23^ When the dichotomous outcome data are sparse (as would be expected in the control group^19^), the asymptotic results can be unreliable; therefore, Fisher's exact tests were used to calculate the exact probability of the possible (2 × 2) tables allowing estimation of the Wald‐test‐associated variance, which corresponds to the ratio of its estimate (log_e_‐odds ratio [OR]) to its standard error. By default, no imputations were used (statistical or otherwise) for the analysis of the primary endpoint, but robustness was assessed via sensitivity analyses which evaluated missing data to explore the effect of departures from the assumption made in the main analysis (missing at random). For the primary outcome, we explored the impact of data not missing at random by a simple imputation of “worst case” (missing = not in remission) and “best case” (missing = “in remission”) scenario.^24^ Continuous outcomes were analysed using a repeated‐measures analysis of covariance and reported the difference between least‐squares means with 95% confidence intervals (CIs). The model included treatment (two levels), time (three to six levels), sex (two levels), and the possible interaction between treatment (group) and time (months) as fixed effects, with the baseline value of the relevant variable as a covariate and participant identifier as a random effect. Model diagnostics were verified using predicted values and residuals were investigated to assess the adequacy of the models.

For the stratified analyses, the missing data were imputed based on a worst‐case assumption; that is, assuming that participants with missing data did not achieve partial or complete remission, *a priori*.^24^ The statistical test of interaction was analysed by comparing whether the net benefit of U‐TURN over standard care varied with subgroup.^25^

Statistical analyses were performed using STATA/IC (StataCorp, College Station, Texas), version 13.1, and the statistical significance level (for superiority) was set at α < 0.05 (two‐tailed). A statistical analysis plan was completed prior to analysis (Appendix S2).

## 3 RESULTS

**Figure 1:**
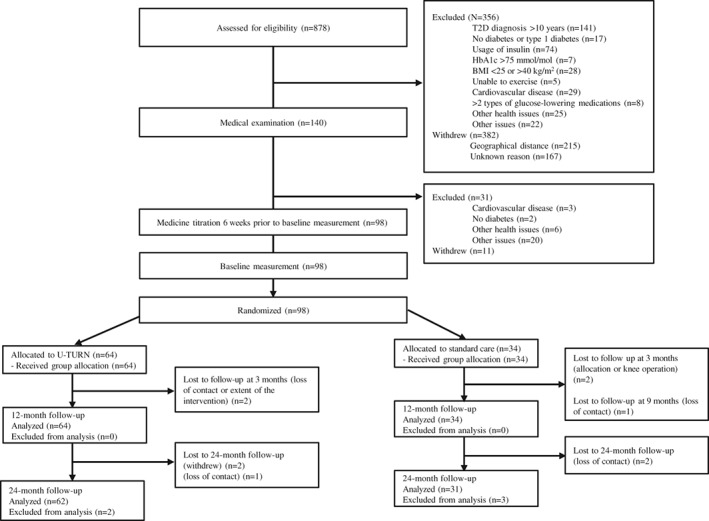
Flow of participants through the study. BMI, body mass index; HbA1c, glycated haemoglobin; T2D, type 2 diabetes

Of the 98 participants enrolled in the trial, 87 (59 in the U‐TURN and 28 in the standard care group) participated in the 24‐month follow‐up assessments (Figure 1). As five participants attending the 12‐month follow‐up had data indicating that they did not reach the criteria for partial or complete T2D remission, they were included in the analysis set for the primary outcome (N_U‐TURN/standard care_ = 62/31). Retention rates did not differ between the groups (*P* = 0.18). The mean (SD) age at baseline was 54.6 (9.0) years, time since diagnosis was 5.1 (2.9) years, and there was an even sex distribution (46.2% women; Table 1). Medication prescription and adherence are described in Table S1 and S2.

**Table 1:** Baseline characteristics of study participants

	U‐TURN group	Standard care group
Demographics		
Number of participants	62	31
Age, y	53.5 (9.2)	56.7 (8.3)
Female, n (%)	29 (47)	14 (45)
T2D duration, years	4.7 (2.8)	5.6 (3.2)
Glycaemic control		
HbA1c, mmol/mol	49.1 (9.1)	49.7 (9.8)
Fasting glucose, mmol/L	8.4 (2.9)	9.1 (3.1)
Median (interquartiles) fasting insulin, pmol/L	113 (82; 162)	116 (65; 176)
2‐h glucose, mmol/L (n = 61/30)	15.4 (4.0)	16.3 (4.2)
Lipids		
Total cholesterol, mmol/L	4.2 (0.9)	4.0 (1.0)
Median (interquartiles) LDL cholesterol, mmol/L	2.4 (1.9; 2.9)	2.1 (1.7; 2.5)
HDL cholesterol, mmol/L	1.2 (0.3)	1.3 (0.4)
Median (interquartiles) triglycerides, mmol/L	1.5 (1.0; 1.9)	1.4 (0.8; 1.8)
Blood pressure		
Systolic, mmHg (n = 59/23)	127 (14)	137 (8)
Diastolic, mmHg (n = 84)	79 (9)	84 (8)
Body composition		
Body mass, kg	95.3 (14.1)	97.6 (15.4)
Body mass index, kg/m^2^	31.5 (3.9)	32.3 (4.4)
Fat mass, kg	35.4 (9.2)	35.8 (10.8)
Lean body mass, kg	57.9 (10.2)	57.9 (10.7)
Abdominal fat mass, kg	4.0 (1.2)	4.1 (1.3)
Physical fitness, physical activity and diet		
VO_2max_, mL O_2_/min (n = 62/30)	2734 (719)	2668 (771)
Relative VO_2max_, mL O_2_/kg/min (n = 62/30)	28.7 (6.6)	26.9 (6.2)
Median (interquartiles) physical activity, MET h/d (n = 58/29)	18 (15; 25)	20 (15; 31)
Median (interquartiles) energy intake, kcal/d (n = 60/25)	2160 (1720; 2574)	2224 (1614; 2637)
Medication usage		
Glucose‐lowering medication, n (%)	62 (98)	31 (100)
Lipid‐lowering medication, n (%)	51 (80)	30 (88)
Blood pressure‐lowering medication, n (%)	31 (48)	19 (56)

Data are mean (SD), unless otherwise indicated.

Abbreviations: HbA1c, glycated haemoglobin; T2D, type 2 diabetes; VO_2max_, maximal oxygen uptake.

### 3.1 Partial and complete T2D remission

**Figure 2:**
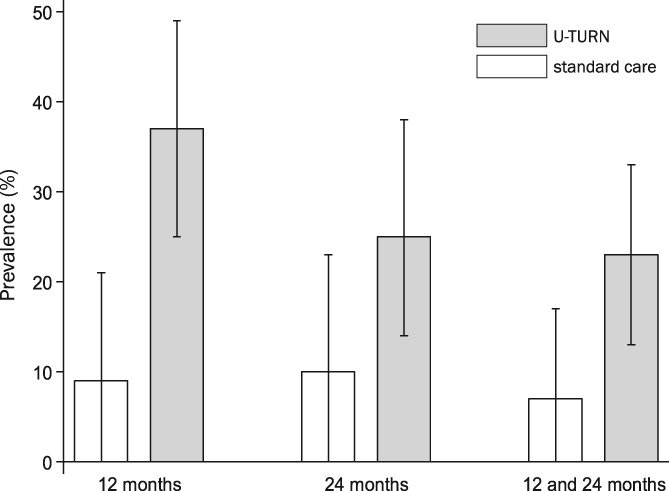
Prevalence of participants with fasting glucose below the diagnostic threshold for type 2 diabetes (T2D [≤6.9 mmol/L]) and glycated haemoglobin (HbA1c) <48 mmol/mol without glucose‐lowering medication at 12 months, 24 months and at both time points (any T2D remission). White bars indicate the standard care group and the grey bars are the U‐TURN group. Mean median values following multiple bootstrap samples (1000 samples with replacement), with the corresponding empirical 95% CIs (ie, 2.5 and 97.5 percentiles) are shown. Data are as‐observed with raw case/denominators for the control group at 12 months (3/31), at 24 months (3/28) and at both time points (2/31) and the corresponding raw case/denominators for the U‐TURN group at 12 months (23/62), at 24 months (15/59) and at both time‐points (14/62)

**Figure 3:**
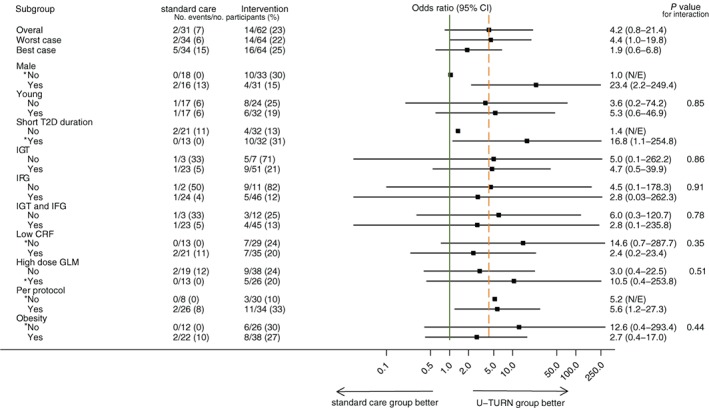
Overall and subgroup (predefined based baseline levels) effects of an intensive lifestyle intervention vs. standard care on the occurrence of full or partial type 2 diabetes (T2D) remission at 24‐month follow‐up in participants with T2D. Young is defined as age < 54 years, based on a median split of age; short T2D duration as <5 years, based on a median split of disease duration, impaired glucose tolerance (IGT) as 2‐hour glucose concentration ≥ 11.1 mmol/L during an oral glucose tolerance test, impaired fasting glucose (IFG) as fasting glucose ≥7.0 mmol/L, low cardio‐respiratory fitness (CRF) as maximal oxygen consumption <2623 mL O_2_/min, based on a median split of age, high‐dose glucose‐lowering medication (GLM) as >1000 mg metformin, and obesity as body mass index >30 kg/m^2^. Per protocol indicates the presence of all the following criteria during the intervention: U‐TURN group: 1) attending at least four (of five) medical consultations, 2) completing ≥70% of all exercise sessions and 3) only receiving the prescribed medications and/or the prescribed combination of medications according to the treatment algorithm; standard care group: 1) attending at least four (of five) medical consultations and 2) only receiving the prescribed medications and/or the prescribed combination of medications according to the treatment algorithm. *Analyses are based on continuity corrected data. N/E, not estimeble

The prevalence of participants reaching sub‐diabetic levels of fasting glucose and HbA1c without glucose‐lowering medication at 12‐ and 24‐month follow‐ups is presented in Figure 2. At 24‐month follow‐up, 14 (23%) and two participants (7%) met the criteria for partial or complete T2D remission in the U‐TURN and standard care groups, respectively (Figure 2). Whereas the odds of remission were more than fourfold higher in the U‐TURN compared with the standard care group, the confidence limits for the OR for remission were wide (95% CI 0.8–21.4; *P* = 0.08). Under the assumption that all participants lost to follow‐up relapsed (did not meet the criteria for remission), the OR for T2D remission was 4.4 (95% CI 1.0–19.8; *P* = 0.048 [Figure 3]). One participant reached complete T2D remission (in the U‐TURN group). Risk ratios for partial and complete T2D remission are presented in Table S3. The number needed‐to‐treat for one partial or complete T2D remission was seven participants, when assuming all participants lost to follow‐up had relapsed.

While the odds for complete or partial T2D remission were higher in the U‐TURN than the standard care group among men and participants with short T2D duration at baseline (Figure 3), no interactions were observed in any of the stratified analyses (Figure 3).

### 3.2 Glycaemic control

Levels of HbA1c increased and fasting insulin levels decreased in both groups (Table 2 and Figure S1), with no between‐group differences. No intervention effects were observed on fasting glucose or 2‐hour glucose (Table 2 and Figure S2).

**Table 2:** Secondary outcomes based on the intention‐to‐treat population from baseline to 24‐month follow‐up

	U‐TURN group				Standard care group				Between group comparison			
	N	Change	LCL 95%	UCL 95%	N	Change	LCL 95%	UCL 95%	MD	LCL 95%	UCL 95%	*P*
Glycaemic control												
HbA1c, mmol/mol	62	2.7	0.8	4.7	31	3.2	0.3	6.1	−0.5	−3.9	3.0	0.80
Fasting insulin, pmol/L	61	−32.1	−44.1	−20.1	27	−37.9	−55.7	−20.1	5.8	−15.7	27.4	0.57
Fasting glucose, mmol/L	61	0.6	0.1	1.1	27	1.2	0.4	1.9	−0.6	−1.5	0.3	0.21
2‐hour glucose, mmol/L	61	−0.7	−1.6	0.1	27	−0.1	−1.4	1.2	−0.6	−2.2	0.9	0.43
Lipids												
Total cholesterol, mmol/L	62	0.5	0.3	0.7	28	0.4	0.1	0.5	0.1	−0.2	0.5	0.50
LDL, mmol/L	62	0.5	0.3	0.6	28	0.4	0.1	0.7	0.1	−0.3	0.4	0.71
HDL, mmol/L	62	0.1	0.0	0.1	28	0.0	−0.1	0.1	0.1	0.0	0.2	0.19
Triglycerides, mmol/L	62	0.1	0.0	0.3	28	0.0	−0.2	0.2	0.1	−0.1	0.4	0.35
Blood pressure												
Systolic, mmHg	54	0.0	−2.4	2.4	21	1.3	−2.7	5.3	−1.3	−6.0	3.4	0.59
Diastolic, mmHg	54	−0.1	−1.8	1.8	21	1.0	−2.0	4.0	−1.0	−4.5	2.5	0.57
Body composition												
Body mass, kg	61	−1.39	−2.89	0.11	28	−0.79	−3.00	1.42	−0.87	−3.28	2.07	0.17
Body mass index, kg/m^2^	61	−0.45	−0.95	0.04	28	−0.31	−1.03	0.41	−0.15	−1.02	0 .73	0.74
Fat mass, kg	61	−1.18	−1.18	0.04	28	0.18	−1.60	1.98	−1.37	−3.53	0.80	0.22
Lean body mass^a^, kg	61	0.09	−1.16	1.03	28	−0.71	−2.16	0.39	1.01	0.00	1.03	0.09
Abdominal fat mass^a^, kg	57	−0.05	−0.34	0.61	27	0.24	−0.32	0.24	0.94	0.82	1.07	0.35
5% body weight reduction^b^, n (%)	16(28)	N/A	N/A	N/A	5(19)	N/A	N/A	N/A	1.7	0.5	5.2	0.37
10% body weight reduction^b^, n (%)	8(14)	N/A	N/A	N/A	2(7)	N/A	N/A	N/A	2.0	0.4	10.1	0.40
Physical fitness												
VO_2max_, mL O_2_/min	61	40.2	−54.0	134.6	25	−219.6	−366.3	−72.9	259.8	85.0	434.6	0.004
Relative VO_2max_, mL O_2_/kg/min	61	1.1	−0.1	2,3	25	−2.2	−4.0	−.4	3.3	1.1	5.5	0.003
Medication												
No glucose‐lowering medication^b^, n (%)	20(34)	N/A	N/A	N/A	4(14)	N/A	N/A	N/A	3.1	0.9	10.1	0.06
No lipid‐lowering medication^b^, n (%)	6(10)	N/A	N/A	N/A	1(4)	N/A	N/A	N/A	2.9	0.3	25.7	0.33
No blood pressure‐lowering medication^b^, n (%)	23(41)	N/A	N/A	N/A	4(17)	N/A	N/A	N/A	3.5	1.1	11.6	0.041
Diet and physical activity												
Energy intake, kcal/kg	55	−134	−266	−2	23	−66	−271	138	−68	−311	176	0.59
Energy expenditure, MET h/d	53	0.6	−1.1	2.2	25	−4.6	−6.4	−1.7	3.9	1.6	6.2	0.001

Data are mean change and upper and lower 95% confidence limits or number (proportions).

Abbreviations: HbA1c, glycated haemoglobin; LCL, lower confidence limit; MD, mean difference; N/A, not available; UCL, upper confidence limit; VO_2max_, maximal oxygen uptake.

a Median change and interquartiles. MD is based on log‐transformed values and reported as ratio of geometric mean.

b Between‐group differences and 95% confidence limits are odds ratios.

### 3.3 Body composition

No differences between the U‐TURN and standard care group were observed in weight, BMI, fat mass, abdominal mass or fat‐free mass from baseline to 24‐month follow‐up (Table 2 and Figure S3). Only a limited number of participants achieved 5% and 10% weight loss (Table 2).

### 3.4 Other secondary outcomes

Physical fitness and physical activity energy expenditure increased more from baseline to 24‐month follow‐up in the U‐TURN group compared to the standard care group (Table 2), and the prevalence of participants not using blood pressure‐lowering medication was higher in the U‐TURN group compared to the standard care group (Table 2). No statistically significant differences in the remaining secondary outcomes were observed (Table 2). More participants experienced musculoskeletal pain in the intervention group compared to the standard care group during the 24 months of follow‐up (*P* = 0.04; Table S4).

### 3.5 *Post hoc* outcomes

Participants meeting the criteria for any T2D remission increased their physical fitness and reduced their abdominal fat mass and total fat mass more from baseline to 24 months than participants not meeting the criteria (Table S5). Remission was associated with a lower decline in physical fitness from 12 to 24 months, but not with changes in self‐reported physical activity or dietary variables (Table S6).

## 4 DISCUSSION

The main finding of the present study was that a 12‐month lifestyle intervention including high exercise volume and dietary counselling may lead to partial T2D remission in participants with well‐controlled T2D of short duration. While the lifestyle intervention increased physical fitness, no differences in indices of glycaemic control, lipidaemia or body composition, were observed between the groups 12 months after completing the intervention.

The rate of partial or complete T2D remission, using the same criteria as in the present study, is extremely low in a representative general diabetes population.^19^ Moreover, the chance of partial T2D remission decreases with time since diagnosis and when glucose‐lowering pharmacological treatment needs intensification.^19^ In a subpopulation with similar duration of T2D duration to that of the population receiving an intensive lifestyle intervention in the present study (4–5 years), Karter et al^19^ observed a considerably lower T2D remission incidence (<2% vs. 23%). However, as a result of the stringent sampling frame, the present study population was probably more willing and motivated to engage in lifestyle change as compared to the background population, which renders direct comparison difficult. In the Look AHEAD study, the rate of any T2D remission was 10.4% after 2 years in the intensive lifestyle intervention group,^6^ which was similar to the T2D remission incidence in the standard care group (7%). This is not surprising given the extensiveness of the treatment regimen in the standard care group^18^; however, several notable differences between the study designs limit direct comparisons. The Look AHEAD trial included participants with longer T2D duration, greater comorbidity burden and older age, all factors that decrease the chance of T2D remission in lifestyle interventions.^6^, ^8^, ^19^ In addition, the remission criteria used in the Look AHEAD study did not stipulate that the prediabetic glucose levels should be maintained without glucose‐lowering medication for a prolonged period of time (ie, ≥1 year). Finally, contrary to the Look AHEAD intensive lifestyle group, the 1 year that followed the termination of the intensive lifestyle intervention in the present study was an observational period (ie, no intervention was provided). In comparison with intensive dietary treatment without exercise intervention, the T2D remission rates (as defined by HbA1c ≤48 mmol/mol without glucose‐lowering medication) in the U‐TURN group were similar 1 year after the intervention^7^; Hence, at 24‐month follow‐up, the DIRECT trial showed partial or full T2D remission in 36.5% of the participants in the intervention group^7^ which is comparable to 34% in the U‐TURN group. Whereas the DIRECT follow‐up study showed a concomitant weight loss, participation in the U‐TURN intervention was not associated with weight loss but with an increase in physical fitness. As T2D remission in the present study was associated with an increase in fitness levels from baseline to 24‐month follow‐up, this may suggest that T2D remission in the present study was obtained by exercise and not only dietary changes. This is highly speculative, however, and the observation needs replication.

Although no quantitative interaction could be detected in the contextual analyses, we observed qualitative sub‐group effects in response to the intervention among participants with shorter T2D duration and among men but not among participants with longer T2D duration and women. In line with previous lifestyle interventions, short T2D duration is a predictor for T2D remission in lifestyle intervention studies.^6^, ^10^, ^26^ This may relate to the underlying pathogenesis of T2D which involves declining β‐cell function.^10^, ^27^ In support of this, Dela et al^26^ observed that, while patients with T2D who had remaining insulin secretory capacity improved their pancreatic β‐cell function in response to a training intervention, patients without remaining secretory capacity did not.^26^ Collectively, this highlights the importance and clinical significance of introducing intensive lifestyle interventions as soon as possible after T2D diagnosis to potentially revert the disease. Contrary to other lifestyle interventions,^6^, ^8^ we observed an intervention effect on T2D remission among men but not among women; however, the significance of that observation is uncertain as the men also had a shorter average T2D duration. In line with other lifestyle intervention studies, the present study indicates that partial T2D remission may be obtainable when participants are engaged in an intensive lifestyle intervention. We extend the existing knowledge by showing that a subgroup of participants was able to maintain glucose levels below the diagnostic criteria without the use of glucose‐lowering medications 1 year after the termination of a lifestyle intervention programme (ie, without maintenance support).

The present study has several limitations. First, some ambiguity exists around the concept of remission. The American Diabetes Association consensus statement defined remission as hyperglycaemia below the diagnostic level for diabetes (partial remission) or returning to the normal level (complete remission) in the absence of active glucose‐lowering therapy. Importantly, these targets should be maintained for at least 1 year.^17^ Moreover, a lowering of fasting glucose or HbA1c alone or in absence of glucose‐lowering medications may not appropriately reflect a decreased risk of complications.^28^ This is further underpinned by our observation that only maximal oxygen uptake and not indices of glycaemic control or body composition were improved. However, it has been suggested that T2D remission attributable to a large diet‐induced weight loss reflects a reversion of T2D pathophysiology,^10^ suggesting clinical relevance. Second, the adherence to glucose‐lowering medical therapy at 24‐month follow‐up was self‐reported, thus was prone to information bias. Consequently, the difference in T2D remission rates may be overestimated if the bias were differential (ie, more frequent in the U‐TURN or standard care group). Third, T2D remission was not a designated outcome in the present study, rendering the present findings exploratory,^18^ consequently, the study was not powered for this analysis and may, therefore, be underpowered. Moreover, although the primary analysis did not reach the level of statistical significance, the worst‐case scenario sensitivity analyses did. This analysis was made under the assumption that participants lost to follow‐up did not achieve any T2D remission. This is a reasonable assumption because the chance of reaching any T2D remission in the background population is very low^19^; therefore, the best‐case scenario (all participants lost to follow‐up achieve any T2D remission) is probably not realistic. As no apparent difference in the point estimate between the primary analysis and the worst‐case scenario was seen, this suggests that the effect of the lifestyle intervention on T2D remission was robust.

In conclusion, an intensive 12‐month exercise‐based lifestyle intervention was associated with nonsignificant partial or complete T2D remission in 23% of all participants compared with only 7% participants receiving standard care. Nevertheless, the difference in this relatively small sample size did not reach statistical significance and further studies on maintaining a lifestyle change are needed.

## CONFLICT OF INTEREST

A.V. was appointed vice president for AstraZeneca's Translational Research and Early Clinical Development during the completion of the study, but remained in the scientific steering committee of this study. R.C. employer, the Parker Institute, Bispebjerg, and Frederiksberg Hospital, is supported by core grant OCAY‐13‐309 from the Oak Foundatian, and he reports receiving personal fees from Abbott, AbbVie, Amgen, Axellus A/S, Bayer HealthCare Pharmaceuticals, Biogen Idec, Bristol‐Myers Squibb, CambridgeWeight Plan, Celgene, Eli Lilly, Hospira, Ipsen, Janssen, Laboratories Expanscience, Merck Sharp & Dohme, Mundipharma, Norpharma, Novartis, Orkla Health, Pfizer, Roche, Rottapharm‐Madaus, Sobi, Takeda and Wyeth, personal fees from employment from Research Unit for Musculoskeletal Function and Physiotherapy, Institute of Sports Science and Clinical Biomechanics, and the University of Southern Denmark, grants pending and grant funding from Axellus A/S, AbbVie, Cambridge Weight Plan, Janssen, Merck Sharp & Dohme, Mundipharma, Novartis and Roche, and being involved in many healthcare initiatives and research that could benefit from wide uptake of this publication including Cochrane, Outcome Measures in Rheumatology, International Dermatology Outcome Measures, RADS and the Grading of Recommendations Assessment, Development and Evaluation Working Group. M.R.‐L. has received personal speakers fees from Novo Nordisk A/S. The remaining authors have no conflict of interest to declare.

## Supporting information

Table S1**.** Self‐reported medication intake at 24 months follow‐up.
Table S2**.** Self‐reported medication adherence at 24 months follow‐up. Participants were asked: How often do you forget to take your prescribed medication?
Table S3. Overall and sub‐group (pre‐defined) effects (as risk ratios with 95% confidence intervals) of an intensive lifestyle intervention vs. standard care on the occurrence of complete or partial type 2 diabetes remission at 24 months follow‐up in patients with type 2 diabetes.
TABLE S4. Adverse events from baseline to 24‐month follow‐up for u‐turn vs standard care groups among participants with non–insulin‐dependent type 2 diabetes.
Table S5. Changes in body composition, cardiorespiratory, physical activity and diet from 0–24 months follow‐up and partial type 2 diabetes remission at 24 months follow‐up.
Table S6. Changes in cardiorespiratory, physical activity and diet from 12–24 months follow‐up and partial type 2 diabetes remission at 24 months follow‐up.
Figure S1. Hemoglobin A_1C_ concentrations for U‐TURN vs. standard care groups among participants with type 2 diabetes, intention‐to‐treat analyses. Data are least squares means derived from mixed linear models, adjusted for the respective sex and baseline levels. Error bars are 95% confidence intervals.
Figure S2. Fasting blood glucose concentrations for U‐TURN vs. standard care groups among participants with type 2 diabetes, intention‐to‐treat analyses. Data are least squares means derived from mixed linear models, adjusted for the respective sex and baseline levels. Error bars are 95% confidence intervals.
Figure S3. Body weight (A). Fat mass (B) and Lean body mass (C) for the U‐TURN (yellow) and StC (green) groups among participants with type 2 diabetes. Intention‐to‐treat analyses. Data are least squared means derived from mixed linear models. Adjusted for the respective sex and baseline levels. Error bars are 95% confidence intervals.

Appendix S1**.** Trial protocol.

Appendix S2**.** Statistical analysis plan.

Appendix S3. CONSORT Check list**.**
