# Supplementary material for: Type 2 diabetes remission 1 year after an intensive lifestyle intervention: A secondary analysis of a randomized clinical trial
Source: Diabetes Obes Metab. 2019 Jun 30;21(10):2257–66. doi: 10.1111/dom.13802 (PMC6772176; doi:10.1111/dom.13802)
Supplement: Supplementary file 1 — Table S1. Self‐reported medication intake at 24 months follow‐up. Table S2. Self‐reported medication adherence at 24 months follow‐up. Participants were asked: How often do you forget to take your prescribed medication? Table S3. Overall and sub‐group (pre‐defined) effects (as risk ratios with 95% confidence intervals) of an intensive lifestyle intervention vs. standard care on the occurrence of complete or partial type 2 diabetes remission at 24 months follow‐up in patients with type 2 diabetes. TABLE S4. Adverse events from baseline to 24‐month follow‐up for u‐turn vs standard care groups among participants with non–insulin‐dependent type 2 diabetes. Table S5. Changes in body composition, cardiorespiratory, physical activity and diet from 0–24 months follow‐up and partial type 2 diabetes remission at 24 months follow‐up. Table S6. Changes in cardiorespiratory, physical activity and diet from 12–24 months follow‐up and partial type 2 diabetes remission at 24 months follow‐up. Figure S1. Hemoglobin A1C concentrations for U‐TURN vs. standard care groups among participants with type 2 diabetes, intention‐to‐treat analyses. Data are least squares means derived from mixed linear models, adjusted for the respective sex and baseline levels. Error bars are 95% confidence intervals. Figure S2. Fasting blood glucose concentrations for U‐TURN vs. standard care groups among participants with type 2 diabetes, intention‐to‐treat analyses. Data are least squares means derived from mixed linear models, adjusted for the respective sex and baseline levels. Error bars are 95% confidence intervals. Figure S3. Body weight (A). Fat mass (B) and Lean body mass (C) for the U‐TURN (yellow) and StC (green) groups among participants with type 2 diabetes. Intention‐to‐treat analyses. Data are least squared means derived from mixed linear models. Adjusted for the respective sex and baseline levels. Error bars are 95% confidence intervals. [file DOM-21-2257-s001.docx]

| **eTable 1: Self-reported medication intake at 24 months follow-up** | | |
| --- | --- | --- |
|  | **U-turn group**  *No (%)*  *N = 59* | **Standard care group**  *No (%)*  *N = 28* |
| **Glucose-lowering medication** |  |  |
| Biguanide | 22 (37) | 14 (50) |
| GLP-1 analogue | 1 (2) | 0 (0) |
| TZD | 1 (2) | 0 (0) |
| Biguanide & GLP-1 analogue | 4 (7) | 9 (32) |
| Biguanide & sulfonylurea | 1 (2) | 0 (0) |
| Biguanide & SGLT-2 inhibitor | 1 (2) | 0 (0) |
| Biguanide & insulin analogue | 1 (2) | 0 (0) |
| Biguanide. GLP-1 analogue & SGLT-2 inhibitor | 0 (0) | 1 (4) |
| Biguanide. sulfonylurea. SGLT-2 inhibitor & DPP-IV inhibitor | 1 (2) | 0 (0) |
| Not taking any medication | 27 (46) | 4 (14) |
| **Lipid-lowering medication** |  |  |
| Statins | 35 (59) | 18 (64) |
| Not taking any medication | 24 (41) | 10 (36) |
| **Blood pressure lowering medication** |  |  |
| ARB | 10 (17) | 9 (32) |
| ARB & thiazide | 3 (5) | 4 (14) |
| ARB. thiazide & β1-selective beta blockers | 1 (2) | 0 (0) |
| ACE inhibitor | 2 (3) | 0 (0) |
| ARB & calcium-channel blocker | 1 (2) | 4 (14) |
| ARB. thiazide & calcium-channel blocker | 2 (3) | 0 (0) |
| Thiazide & calcium-channel blocker | 2 (3) | 1 (4) |
| Not taking any medication | 38 (64) | 10 (36) |
| **Other medication** |  |  |
| ASA | 7 (12) | 4 (14) |
| Estrogen & progesterone | 1 (2) | 0 (0) |
| Cytostatic | 1 (2) | 0 (0) |
| Aldosterone antagonist | 0 (0) | 2 (7) |
| Quinine | 0 (0) | 1 (4) |
| Proton pump inhibitor | 1 (2) | 0 (0) |
| Thiazide & potassium chloride (diuretic) | 0 (0) | 1 (4) |
| Loop diuretic & calcium chloride | 1 (2) | 0 (0) |

Abbreviations: GLP 1. glucagon like peptide 1; TZD. thiazolidinedione. SGLT-2. sodium-glucose co-transporter-2; DPP-IV. dipeptidyl peptidase 4; ARB. angiotensin receptor blocker. ACE. angiotensin converting enzyme; ASA. acetylsalicylic acid.

| **eTable 2: Self-reported medication adherence at 24 months follow-up.**  **Participants were asked: How often do you forget to take your prescribed medication?** | | |
| --- | --- | --- |
|  | **Lifestyle group**  *No (%)*  *N = 59* | **Standard care group**  *No (%)*  *N = 28* |
| **Glucose-lowering medication. self-reported medical adherence** |  |  |
| Several times per week | 1 (2) | 1 (4) |
| Once a week | 3 (5) | 1 (4) |
| Several times per month | 1 (2) | 4 (14) |
| Once a month | 6 (10) | 6 (21) |
| Never | 21 (36) | 12 (20) |
| Not taking any medication | 27 (46) | 4 (14) |
| **Lipid-lowering medication. self-reported medical adherence** |  |  |
| Several times per week | 2 (3) | 2 (7) |
| Once a week | 6 (10) | 0 (0) |
| Several times per month | 2 (3) | 4 (14) |
| Once a month | 5 (8) | 4 (14) |
| Never | 20 (34) | 8 (29) |
| Not taking any medication | 24 (41) | 10 (36) |
| **Blood pressure lowering medication. self-reported medical adherence** |  |  |
| Several times per week | 0 (0) | 1 (4) |
| Once a week | 2 (3) | 0 (0) |
| Several times per month | 0 (0) | 4 (14) |
| Once a month | 6 (10) | 5 (18) |
| Never | 13 (22) | 8 (29) |
| Not taking any medication | 38 (64) | 10 (36) |

| **eTable 3** Overall and sub-group (pre-defined) effects (as risk ratios with 95% confidence intervals) of an intensive lifestyle intervention vs. standard care on the occurrence of complete or partial type 2 diabetes remission at 24 months follow-up in patients with type 2 diabetes. | | | | | | | | | | |
| --- | --- | --- | --- | --- | --- | --- | --- | --- | --- | --- |
|  | **U-TURN** | | | **Standard care** | | |  |  |  |  |
| **T2D Remission** | **YES (N)** | **NO (N)** | **Total (N)** | **YES (N)** | **NO (N)** | **Total (N)** | ***RR*** | ***LCL95%*** | ***UCL95%*** | ***Interaction:*** |
|  |  |  |  |  |  |  |  |  |  |  |
| **All patients** | 14 | 48 | 62 | 2 | 29 | 31 | 3.50 | 0.87 | 14.10 |  |
| **’Best case’ (all missing in remission)** | 16 | 48 | 64 | 5 | 29 | 34 | 1.70 | 0.62 | 4.69 |  |
| **’Worst case’ (all missing relapse)** | 14 | 50 | 64 | 2 | 32 | 34 | 3.72 | 1.01 | 13.68 |  |
|  |  |  |  |  |  |  |  |  |  |  |
| **Male** |  |  |  |  |  |  |  |  |  | **p-value** |
| YES^#^ | 10 | 23 | 33 | 0 | 18 | 18 | 16.45 | 2.01 | 134.51 |  |
| NO | 4 | 27 | 31 | 2 | 14 | 16 | 1.03 | N/E | N/E |  |
|  |  |  |  |  |  |  |  |  |  |  |
| **Young** |  |  |  |  |  |  |  |  |  | **0.88** |
| YES | 6 | 26 | 32 | 1 | 16 | 17 | 3.19 | 0.69 | 14.67 |  |
| NO | 8 | 24 | 32 | 1 | 16 | 17 | 4.25 | 0.15 | 120.93 |  |
|  |  |  |  |  |  |  |  |  |  |  |
| **Short T2D duration** |  |  |  |  |  |  |  |  |  |  |
| YES^#^ | 10 | 22 | 32 | 0 | 13 | 13 | 15.06 | 1.10 | 205.62 |  |
| NO | 4 | 28 | 32 | 2 | 19 | 21 | 1.31 | N/E | N/E |  |
|  |  |  |  |  |  |  |  |  |  |  |
| **Impaired glucose tolerance (IGT) (N=84)** |  |  |  |  |  |  |  |  |  | **0.67** |
| YES | 9 | 42 | 51 | 1 | 22 | 23 | 4.06 | 0.58 | 28.40 |  |
| NO | 5 | 2 | 7 | 1 | 2 | 3 | 2.14 | 0.24 | 19.52 |  |
|  |  |  |  |  |  |  |  |  |  |  |
| **Impaired fasting glucose (IFG) (N=83)** |  |  |  |  |  |  |  |  |  | **0.84** |
| YES | 5 | 41 | 46 | 1 | 23 | 24 | 2.61 | 0.04 | 177.79 |  |
| NO | 9 | 2 | 11 | 1 | 1 | 2 | 1.64 | 0.49 | 5.50 |  |
|  |  |  |  |  |  |  |  |  |  |  |
| **IGT and IFT (N=83)** |  |  |  |  |  |  |  |  |  | **0.56** |
| YES | 5 | 40 | 45 | 1 | 22 | 23 | 2.56 | 0.04 | 160.16 |  |
| NO | 3 | 9 | 12 | 1 | 2 | 3 | 0.75 | 0.46 | 1.21 |  |
|  |  |  |  |  |  |  |  |  |  |  |
| **Low cardio-respiratory fitness** |  |  |  |  |  |  |  |  |  | **0.32** |
| YES | 7 | 28 | 35 | 2 | 19 | 21 | 2.10 | 0.30 | 14.97 |  |
| NO^#^ | 7 | 22 | 29 | 0 | 13 | 13 | 11.14 | 0.76 | 164.08 |  |
|  |  |  |  |  |  |  |  |  |  |  |
| **High dose of glucose lowering medications** |  |  |  |  |  |  |  |  |  | **0.43** |
| YES^#^ | 5 | 21 | 26 | 0 | 13 | 13 | 8.50 | 0.47 | 155.51 |  |
| NO | 9 | 29 | 38 | 2 | 17 | 19 | 2.25 | 0.49 | 10.33 |  |
|  |  |  |  |  |  |  |  |  |  |  |
| **Per protocol (0-12 months)** |  |  |  |  |  |  |  |  |  |  |
| YES | 11 | 23 | 34 | 2 | 24 | 26 | 4.21 | 1.17 | 15.18 |  |
| NO^#^ | 3 | 27 | 30 | 0 | 8 | 8 | 1.43 | N/E | N/E |  |
|  |  |  |  |  |  |  |  |  |  |  |
| **Obesity** |  |  |  |  |  |  |  |  |  | 0.42 |
| YES | 8 | 30 | 38 | 2 | 20 | 22 | 2.32 | 0.47 | 11.37 |  |
| NO^#^ | 6 | 20 | 26 | 0 | 12 | 12 | 9.77 | 0.44 | 215.97 |  |
|  |  |  |  |  |  |  |  |  |  |  |

T2D = type 2 diabetes, IGT indicates Impaired glucose tolerance, IFG indicates Impaired Fasting Glucose, IGT and IFG indicates Impaired glucose tolerance and fasting blood glucose, CRF indicates Cardio-Respiratory Fitness, GLM indicates Glucose Lowering Medications, LCL95% indicates Lower 95% Confidence limit, UCL95% indicates Upper 95% Confidence limit, N/E indicates Not estimable (Fisher’s exact p-value = 1.00), # indicates continuity correction

| **eTABLE 4** Adverse Events from Baseline to 24-Month Follow-up for U-TURN vs Standard Care Groups Among Participants With Non–Insulin-Dependent Type 2 Diabetes | | | | | | |
| --- | --- | --- | --- | --- | --- | --- |
|  | U-TURN (N=64) | |  | Standard Care (n = 34) | | p-value |
|  | N | % |  | N | % |  |
| **Serious Adverse Events** |  |  |  |  |  |  |
| Total | 1 | 2 |  | 0 | 0 | 1.00 |
| Death | 0 | 0 |  | 0 | 0 | N/E |
| Severe hypoglycemia^a^ | 0 | 0 |  | 0 | 0 | N/E |
|  |  |  |  |  |  |  |
| **Adverse events** |  |  |  |  |  |  |
| Mild hypoglycemia^b^ | 9 | 14 |  | 1 | 3 | 0.16 |
| Any musculoskeletal pain or discomfort^c^ | 18 | 28 |  | 3 | 9 | 0.04 |
| Gastrointestinal problems^d^ | 14 | 22 |  | 10 | 29 | 0.45 |
| Mild hypotension | 4 | 6 |  | 2 | 6 | 1.00 |
| Insomnia | 0 | 0 |  | 1 | 3 | N/E |
| Peripheral edema | 5 | 8 |  | 2 | 6 | 1.00 |
| Xerostomia | 4 | 8 |  | 1 | 3 | 0.67 |
| Fatigue | 2 | 3 |  | 0 | 0 | 0.54 |
|  |  |  |  |  |  |  |

^a^Episodes requiring medical intervention, third party assistance or plasma glucose < 54mg/dL,.

^b^Signs of hypoglycemia include sweating, dizziness, increased non-exercise heart rate, hunger, feeling uncomfortable and confusion.

^c^Any musculoskeletal pain or discomfort reported including an immediate sensation of discomfort, pain, or loss of functioning during exercise or musculoskeletal pain causing cessation of exercise for >=7 consecutive days.

^d^Includes nausea, vomiting, diarrhea, constipation, hypogeusia and dyspepsia

P-value derived from a Fisher’s exact test

N/E not estimable

| **eTable 5** Changes in body composition, cardiorespiratory, physical activity and diet from 0-24 months follow-up and partial type 2 diabetes remission at 24 months follow-up | | | | | | | | | | | | |
| --- | --- | --- | --- | --- | --- | --- | --- | --- | --- | --- | --- | --- |
|  |  |  |  |  |  |  |  |  |  |  |  |  |
|  | **No remission** | | |  | **Remission** | | |  |  |  |  |  |
|  |  |  |  |  |  |  |  |  |  |  |  |  |
| N | 77 |  |  |  | 16 |  |  |  |  |  |  |  |
|  |  |  |  |  |  |  |  |  |  |  |  |  |
|  | *Change* | *LCL95%* | *UCL95%* |  | *Change* | *LCL95%* | *UCL95%* |  | *MD* | *LCL95%* | *UCL95%* | *P-value* |
| Body mass, kg | -1.1 | -2.9 | 0.7 |  | -2.7 | -6.5 | 1.0 |  | -1.6 | -5.8 | 2.5 | 0.44 |
| Body mass index, kg/m^2^ | -0.4 | -1.0 | 0.2 |  | -0.9 | -2.1 | 0.3 |  | -0.5 | -1.9 | 0.8 | 0.46 |
| Fat mass, kg | -0.3 | -1.4 | 0.9 |  | -4.7 | -7.2 | -2.3 |  | -4.5 | -7.2 | -1.8 | 0.001 |
| Abdominal fat mass, kg¤ | 0.2 | -0.3 | 1.0 |  | -0.3 | -1.7 | 0.0 |  | 0.8 | 0.7 | 0.9 | 0.01 |
| Lean body mass, kg | -1.1 | -2.8 | 0.7 |  | -0.3 | -4.0 | 3.4 |  | 0.1 | -1.7 | 1.7 | 0.84 |
| 5% body weight reduction (n=69/16)^†^ | 16 (23.1) | N/A | N/A |  | 5 (32.1) | N/A | N/A |  | 1.5 | 0.4 | 5.4 | 0.53 |
| 10% body weight reduction (n=69/16) ^†^ | 5 (7.2) | N/A | N/A |  | 5 (32.1) | N/A | N/A |  | 5.8 | 1.4 | 25.0 | 0.02 |
| VO_2peak_, ml O_2_/min | -68.8 | -150.9 | 13.4 |  | 155.7 | -13.1 | 324.5 |  | 224.5 | 35.7 | 413.3 | 0.02 |
| Relative VO_2peak_, ml O_2_/kg/min | -0.3 | -1.3 | 0.8 |  | 2.9 | 0.8 | 5.0 |  | 3.2 | 0.8 | 5.6 | 0.01 |
| Physical activity energy expenditure (MET hours/day) | -1.4 | -2.8 | 0.1 |  | 1.2 | -1.9 | 4.3 |  | 2.5 | -0.9 | 6.0 | 0.14 |
|  |  |  |  |  |  |  |  |  |  |  |  |  |
| N | 62 |  |  |  | 16 |  |  |  |  |  |  |  |
| Energy intake (kcal) | -88 | -235 | 59 |  | -155 | -444 | 135 |  | -66 | -391 | 258 | 0.69 |
| Carbohydrate (E%) | 2 | 0 | 3 |  | 3 | 0 | 6 |  | 2 | -2 | 4 | 0.38 |
| Fat (E%) | 2 | 1 | 3 |  | 2 | 0 | 4 |  | 0 | -3 | 3 | 0.95 |
| Protein (E%) | 1 | 1 | 2 |  | 1 | -1 | 2 |  | -1 | -2 | 1 | 0.48 |
| Alcohol (E%) | -4 | -5 | -4 |  | -4 | -5 | -3 |  | 0 | -1 | 2 | 0.54 |
|  |  |  |  |  |  |  |  |  |  |  |  |  |

Data are changes from baseline to 24 months follow-up with 95% confidence intervals adjusted for the respective baseline values

MD; Mean difference, LCL; lower confidence limit, UCL; upper confidence limit, N/A; not available, E%; energy percentage

† Numbers (%) and change is Odds ratio with 95% Wald confidence limits and Fisher’s exact p-values at 24-month follow-up

¤ Median change and interquartile ranges. MD is based on log-transformed values and reported as ratio of geometric mean.

| **eTable 6** Changes in cardiorespiratory, physical activity and diet from 12-24 months follow-up and partial type 2 diabetes remission at 24 months follow-up | | | | | | | | | | | | |
| --- | --- | --- | --- | --- | --- | --- | --- | --- | --- | --- | --- | --- |
|  |  |  |  |  |  |  |  |  |  |  |  |  |
|  | **No remission** | | |  | **Remission** | | |  |  |  |  |  |
|  |  |  |  |  |  |  |  |  |  |  |  |  |
| N | 64 |  |  |  | 16 |  |  |  |  |  |  |  |
|  |  |  |  |  |  |  |  |  |  |  |  |  |
|  | *Change* | *LCL95%* | *UCL95%* |  | *Change* | *LCL95%* | *UCL95%* |  | *MD* | *LCL95%* | *UCL95%* | *P-value* |
| VO_2peak_, ml O_2_/min | -341.9 | -409.1 | -274.6 |  | -150.9 | -287.1 | -14.6 |  | 191.0 | 38.0 | 343.9 | 0.01 |
| Relative VO_2peak_, ml O_2_/kg/min | -4.8 | -5.6 | -4.0 |  | -3.4 | -5.1 | -1.8 |  | 1.5 | -0.5 | 3.1 | 0.15 |
| Physical activity energy expenditure (MET hours/day) | -1.0 | -2.3 | 0.2 |  | -0.9 | -3.5 | 1.8 |  | 0.2 | -2.8 | 3.1 | 0.92 |
|  |  |  |  |  |  |  |  |  |  |  |  |  |
| N | 60 |  |  |  | 16 |  |  |  |  |  |  |  |
| Energy intake (kcal/day) | 91 | -46 | 228 |  | 77 | -188 | 342 |  | -13 | -312 | 285 | 0.93 |
| Carbohydrate (%) | 1 | 0 | 3 |  | 2 | -1 | 5 |  | 1 | -2 | 4 | 0.61 |
| Fat (%) | 3 | 2 | 4 |  | 3 | 1 | 5 |  | 0 | -3 | 2 | 0.93 |
| Protein (%) | 1 | 0 | 2 |  | 0 | -1 | 1 |  | -1 | -2 | 1 | 0.18 |
| Alcohol (%) | -5 | -5 | -4 |  | -4 | -5 | -3 |  | 1 | -1 | 2 | 0.91 |
|  |  |  |  |  |  |  |  |  |  |  |  |  |

Data are changes from 12 to 24 months follow-up with 95% confidence intervals adjusted for the respective values at 12 months follow-up

MD; Mean difference, LCL; lower confidence limit, UCL; upper confidence limit, N/A; not available, , E%; energy percentage

† Numbers (%) and change is Odds ratio with 95% Wald confidence limits and Fisher’s exact p-values at 24-month follow-up

¤ Median change and interquartile ranges. MD is based on log-transformed values and reported as ratio of geometric mean.

**eFigure 1** Hemoglobin A_1C_ concentrations for U-TURN vs. standard care groups among participants with type 2 diabetes, intention-to-treat analyses. Data are least squares means derived from mixed linear models, adjusted for the respective sex and baseline levels. Error bars are 95% confidence intervals

**eFigure 2** Fasting blood glucose concentrations for U-TURN vs. standard care groups among participants with type 2 diabetes, intention-to-treat analyses. Data are least squares means derived from mixed linear models, adjusted for the respective sex and baseline levels. Error bars are 95% confidence intervals


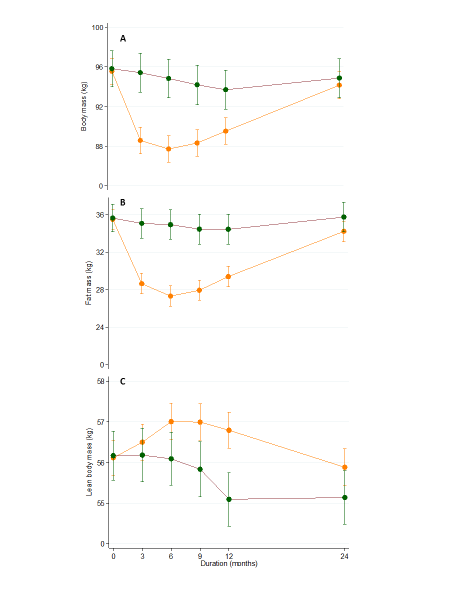


**eFigure 3** Body weight (A). Fat mass (B) and Lean body mass (C) for the U-TURN (yellow) and StC (green) groups among participants with type 2 diabetes. intention-to-treat analyses. Data are least squared means derived from mixed linear models. adjusted for the respective sex and baseline levels. Error bars are 95% confidence intervals
