# Supplementary material for: Type 2 diabetes remission 1 year after an intensive lifestyle intervention: A secondary analysis of a randomized clinical trial
Source: Diabetes Obes Metab. 2019 Jun 30;21(10):2257–66. doi: 10.1111/dom.13802 (PMC6772176; doi:10.1111/dom.13802)
Supplement: Supplementary file 2 — Appendix S1. Trial protocol. [file DOM-21-2257-s002.pdf]

STATISTICAL ANALYSIS PLAN (SAP)

Partial or complete remission following an intensive lifestyle treatment compared to conventional multi-factorial care in type 2 diabetes patients: 2-year follow-up of the randomized, non-blinded, parallel group U-TURN trial

Version 1.5 (December 18th, 2017)

SAP authors:

Mathias Ried-Larsen, MSc, PhD, Principal investigator, Centre for Physical Activity Research, Rigshospitalet, Denmark
Robin Christensen, MSc, PhD, Senior Biostatistician, Professor of Clinical Epidemiology, adj.; Musculoskeletal Statistics Unit, The Parker Institute, Bispebjerg, Frederiksberg Hospital, Copenhagen, Denmark
Kristian Karstoft, MD, PhD, Group leader, Centre for Physical Activity Research, Rigshospitalet, Denmark

Trial registration: NCT02417012

Background and aim: In 2015-2016 we completed the U-TURN lifestyle intervention program aiming at improving glycemic control and induce reductions in glucose-lowering medications. The primary outcome measure of the U-TURN trial was change in HbA1c from baseline to 12 month follow-up, with the hypothesis that lifestyle intervention was equally efficient in maintaining glycemic control compared to standard care. It was found that a 12-month intensive lifestyle intervention was not equally effective to maintain the glycemic control compared to standard clinical care in patients with type 2 diabetes (T2D) diagnosed < 10 years, but rather show potential benefits of an intensive lifestyle intervention on glycemic control[1]. The trial moreover showed that the targeted level of glycemic control could be obtained with considerably less glucose-lowering medication for the participants in the intensive lifestyle group[1]. In fact, 56% of the participants in the intensive lifestyle group discontinued their glucose-lowering medications while nearly normalizing their HbA1c, suggesting T2D remission. However, remission cannot be evaluated based on discontinuation of medications alone, and an acceptable glycemic control without medications should be maintained for at least 12 months[2]. In the previous article, reductions and discontinuations of glucose-lowering medications were compared between groups. This was possible as the medical algorithm was standardized and administered by a blinded-endocrinologist. Following the 12-months trial, the participants were referred back to the general practitioner, rendering it possible that different algorithm and classes of medications was used in the 1 year observational follow-up period. Thus, it may be impossible to recalculate the long-term effects of the interventions on reductions and discontinuations of glucose lowering medications.

The aim of this study is to report the long-term effects on partial or complete T2D remission and other important outcomes following the U-TURN intervention. Specifically, we aim to report the effects of the intervention 24 months following the initiation of the U-TURN intervention. This extension study is designed as a pragmatic superiority trial.

ANALYSIS OBJECTIVES AND OUTCOMES

Primary objective:

The primary objective is to test the hypothesis that the investigational intervention (U-TURN) is superior in inducing partial or complete T2D remission compared to a comparator (standard diabetes care) 12 months following the primary outcome assessment (at 24 months from baseline).

Secondary objectives:

Secondary objectives include reporting the long-term effects (24 months from baseline) on the secondary outcomes (listed below with specific time-points) based on a superiority approach. Moreover, for the complete or partial T2D remission, we will explore possible effect modifiers among contextual factors (i.e. subgroup analyses) from variables assessed at baseline: sex, diabetes duration, age, cardio-respiratory fitness, the need for glucose-lowering medications, and type 2 diabetes phenotype. As a time-dependent variable, we will investigate the subgroup effect on participants adherent (*per protocol*) to either intervention (StC or U-TURN) from time-point 0 (baseline) to time-point 12 months (termination of the intervention).

A schematic overview of the intervention and follow-up period is depicted in **Figure 1**.

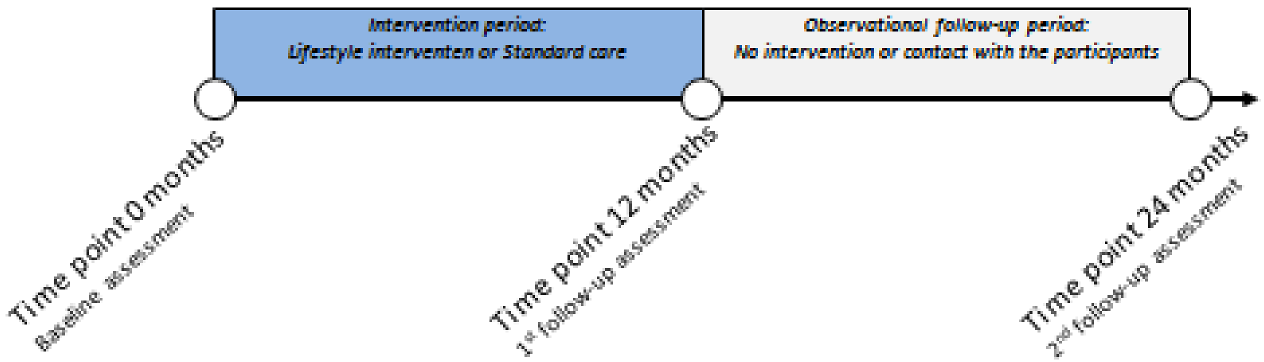

Figure 1: Schematic overview of the study. Blue is the intervention period and the gray is the observational follow-up.

ANALYSIS SET, STUDY POPULATION AND SUBGROUPS

This study will be based on the set of participants that is as close as possible to the ideal implied by the intention-to-treat (ITT) principle; i.e. assessing the effect of a "treatment policy" that is, the planned treatment regimen rather than the actual treatment given. ITT has the consequence that participants allocated to a treatment group should be followed up, assessed and analyzed as members of that group irrespective of their compliance with the planned course of treatment. The 'Full Analysis Set' will be derived from the set of all randomized participants by minimal and justified elimination of participants[1]; participants allocated to a treatment group (U-TURN or StC) will be followed up, assessed and analyzed as members of that group irrespective of their compliance to the planned course of treatment[3].

All participants randomly allocated to either the U-TURN or the StC group unless they explicitly declined further participation between time points 0 and 12 months (N=5) invited to participate in the follow-up assessments at time-point 24 months. Thus, a total of 93 participants (95% of the original sample) have been invited to participate in the follow-up procedures. Data for this report will entail already collected data[1,3] and new data collected at time-point 24 months.

The primary analysis of the primary endpoint will be performed according to the ITT-principle, in terms of analyzing all participants according to the treatment by which they were allocated, regardless of compliance to the treatment; no statistical imputations will replace missing data (*data as-observed).*

Subgroup analyses on the primary end-point will be performed according to sex, diabetes duration, age, diabetes phenotype, glucose-lowering medications and adherence to the

intervention (from time point 0 to 12 months). For the subgroup analyses the missing data will be imputed based on a worst-case assumption; i.e. assuming that patients with missing data did not achieve partial or complete remission[4].

OUTCOMES AND COVARIATES

Primary endpoint: The primary outcome of this trial extension, a comparison between the proportions of participants in each group, who had obtained partial or complete type 2 diabetes remission at 24-months follow-up. Group differences will be analyzed as odds ratios[5], but will be presented and interpreted as risk ratios and risk difference in order to facilitate interpretation [6].

Partial or complete type 2 diabetes remission is based on the definition of partial remission where complete remission is included. Thus, partial or complete remission is defined as the presence at time-point 12 and 24 months of all three criteria ([Fasting glucose ≤6.9 mmol/L] AND HbA1c <48 mmol/mol AND No utilizations of glucose lowering medications)[2,7]. Thus, the primary outcome of this study is dichotomous (0= did not obtain remission or 1=did obtain remission according to the criteria described above).

Pharmaceutical intervention (yes/no) at time-point 24 months will be assessed based on the patient's lists (brought to the 24-month follow-up assessment) or reports of medications and an interview with a nurse. If no glucose lowering medications where listed and the nurse, based on the interview, confirmed that no glucose-lowering medications were utilized, then the patient was categorized as 'not receiving glucose-lowering therapy'. Otherwise, the participant would be categorized as 'receiving glucose-lowering therapy'.

Secondary outcome measures:

1. Between-group difference in changes in glycemic control (HbA1c, fasting insulin, fasting glucose, 2-hour OGTT glucose) from time points 0 to 24 months
2. Between-group difference in changes in metabolic markers of lipidemia (triglycerides, total cholesterol, low-density lipoprotein, high-density lipoprotein) from time points 0 to 24 month follow-up
3. Between-group difference in changes in systolic and diastolic blood pressure from time points 0 to 24 month follow-up
4. Between-group difference in changes in cardio-respiratory fitness from time points 0 to 24 month follow-up
5. Between-group difference in changes in body composition (weight, BMI, fat mass (total, android), lean body mass) from time points 0 to 24-month follow-up
6. Between-group difference in changes in body composition measurements (weight, BMI, fat mass (total, android), lean body mass) from time points 0 to 24 months follow-up
7. Proportion of patients who obtain a 5% or 10% weight loss at time point 24 months follow-up. Obtaining the weight loss of 5 and 10%, respectively, will be expressed in a dichotomous variable (YES=did obtain the pre-specified weight loss or NO= did NOT obtain the pre-specified weight loss).
8. Changes in self-reported physical activity energy expenditure from time point 0 to 24 months follow-up
9. Changes in self-reported energy intake from time point 0 to 24 months follow-up
10. Complete type 2 diabetes remission: Complete type 2 diabetes remission will be a composite defined as the presence at time-point 12 and 24 months of all three criteria: 1) Fasting glucose <5.6 mmol/L, 2) HbA1c<39 mmol/mol, 3) no utilization of glucose lowering

medications[2,7]. Thus, the outcome is dichotomous (0= did not obtain partial remission or 1=did obtain complete remission according to the criteria described above).

11. Without need for glucose lowering medication at time point 24 months according to the protocol. The variable is binary and defined as 0= no need (HbA1c=<48 mmol/mol and no glucose-lowering medications) or 1= need for glucose lowering medications (HbA1c>48 mmol/mol with or without glucose-lowering medication).
12. Without need for lipid lowering medication at time point 24 months according to the protocol. The variable is binary and defined as 0= no need (LDL=<2.5 mmol/L and triglyceride=<5.0 mmol/L and no lipid lowering medications) or 1=need for lipid lowering medications (LDL>2.5 mmol/L and triglyceride>5.0 mmol/L and with/without lipid lowering medications).
13. Without need for blood pressure lowering medication at time point 24 months according to the protocol. The variable is binary and defined as 0= no need (systolic blood pressure=<130 mm/Hg and diastolic blood pressure=<80 mmHg and no blood pressure lowering medications) or 1= need for blood pressure lowering medications (systolic blood pressure>130 mm/Hg and diastolic blood pressure>80 mmHg and with or without blood pressure lowering medications).
14. Adverse events; In the study, we have adopted the ICH definition of AE (E2A). To monitor the AE we have employed a passive surveillance of AE. I.e. we have relied on the participants to actively report all AE's to our medical team. In order to ensure that the participants will likely self-report AE, two procedures have been employed;
    - In the consent form and participant information material, the participants have been primed as the documents state that AE's can occur and which AE's are expected.

- Following the data collection at time point 24-months, all participants were interviewed (semi-structured) by a nurse about possible AE's (discomforts and serious adverse events (see definition below) retrospectively). The study nurse has reported all AE's in the patient report as well, with an indications if they are likely related to the treatments. In case of serious AE's, the Ethical Committee of the Capital Region of Denmark, has been notified by the medical team.

An AE is defined as; "*An adverse event (AE) can therefore be any unfavourable and unintended sign (including an abnormal laboratory finding, for example), symptom, or disease temporally associated with the use of a medicinal product, whether or not considered related to the medicinal product (*http://www.ich.org/fileadmin/Public_Web_Site/ICH_Products/Guidelines/Efficacy/E2A/Step4/E2A_Guideline.pdf*).*"

A serious AE is defined as; *"[...] any untoward medical occurrence that at any dose: * results in death, * is life-threatening, NOTE: The term "life-threatening" in the definition of "serious" refers to an event in which the patient was at risk of death at the time of the event; it does not refer to an event which hypothetically might have caused death if it were more severe. * requires inpatient hospitalisation or prolongation of existing hospitalisation, * results in persistent or significant disability/incapacity, or * is a congenital anomaly/birth defect (*[http://www.ich.org/fileadmin/Public_Web_Site/ICH_Products/Guidelines/Efficacy/E2A/Step4/E2A_Guideline.pdf)."

HANDLING OF MISSING DATA AND OTHER DATA CONVENTIONS

Per default, no imputations will be used (statistical or otherwise) for the analysis of the primary endpoint.

On the basis of using repeated-measures generalized linear mixed models, we have thus chosen the *as observed*-approach as our base-case (i.e. no imputations needed). Thus, the full analysis set contains data from all the patients randomized[8]. These models are valid if data is missing at random (MAR)[9]. Because bias can occur in subtle or unknown ways and its effect is not measurable directly, we will evaluate the robustness of the results and primary conclusions of the trial, by performing sensitivity analyses to explore the effect of departures from the assumption made in the main analysis (MAR). For sensitivity, we will apply a worst-case scenario data imputation to replace missing data at 24 months.

Robustness is a concept that refers to the sensitivity of the overall conclusions to various limitations of the data, assumptions, and analytic approaches to data analysis. Conceptually, robustness will imply that the treatment effect and primary conclusions of the trial are not substantially affected when analyses are carried out based on analysis populations. For the primary outcome (partial or complete remission; 24 months) we will attempt to explore the impact of data not missing at random by a simple imputation of "worst case" (missing = Not in remission) and "best case" (missing = "In remission") scenario[4].

STATISTICAL METHODOLOGY

All data will be collected longitudinally. All data (except self-report data on physical activity and dietary registration) was entered into the database twice, by two separate staff members. Inconsistencies were corrected using the original registrations and the participant journals.

SAMPLE SIZE CONSIDERATIONS

The sample size is this study was based on what was considered feasible, within the local context, enabling up to 120 participants to be enrolled in the trial period (April 29th, 2015 to August 17th, 2017). The sample size was truncated at 120 participants or the N reached at the end of recruitment period. To increase the sensitivity to the U-TURN intervention it was decided to randomize the participants in a 2:1 fashion. Retention at time-point 12 months was 95% and was distributed according to the original randomization procedure by group. As this statistical analysis plan is drafted prior to knowledge about retention and prevalence of complete T2D remission time point 24 months, different power analyses scenarios are outlined in **Table 1**. Based on a chi-squared test (superiority), the statistical power analyses indicated that we would be able to detect a between-group difference (with 80% power and α=0.05) of 20-25% (Number Needed to Treat: 4 to 5 patients) in the proportion of participants achieving remission of T2D, even with a low retention (79.5%) if we have at least 48 participants in the U-TURN group and 25 in the StC group.

Table 1 *Statistical power analyses for a 2-sample proportions X^2-test.*

Scenario	StC – prevalence (%)	RD	Power (1-β)	N U-TURN	N StC
1	5	0.25	0.88	62	31
2	5	0.20	0.77	62	31
3	5	0.15	0.61	62	31
4	1	0.25	0.94	62	31
5	1	0.20	0.87	62	31
6	1	0.15	0.76	62	31
7	5	0.25	0.84	55	28
8	5	0.20	0.72	55	28
9	5	0.15	0.56	55	28
10	1	0.25	0.91	55	28
11	1	0.20	0.84	55	28
12	1	0.15	0.72	55	28
13	5	0.25	0.80	48	25
14	5	0.20	0.67	48	25
15	5	0.15	0.51	48	25
16	1	0.25	0.88	48	25
17	1	0.20	0.80	48	25
18	1	0.15	0.67	48	25

The analyses are based on minimum risk differences of interest at time-point 24 months in complete T2D remission, expected retention rates and standard care prevalence of complete T2D remission.
RD; risk-difference, N; number of participants, StC; Standard care

Primary endpoint

The primary endpoint is based upon group difference in the prevalence of participants obtaining partial or complete T2D remission at time-point 24 months (see definition above). The analysis of the primary outcome is based on a superiority approach; i.e., we expect that the U-TURN intervention is superior in inducing complete or partial T2D remission compared to standard care at time point 24 months. The primary endpoint will be analyzed using logistic regression. The models include fixed factors for group (2 levels) and sex (2 levels – male and female). For the purpose of interpretation, the odds ratio will be converted to risk ratios for the purpose of interpretation[5,6].

Secondary endpoints

Continuous secondary endpoints are based upon group differences in the change from time-point 0 to 24 months according to intention-to-treat. The analyses of the secondary outcomes are based on a superiority approaches. I.e. we expect that the U-TURN intervention is superior in improving markers of glycemic control (reducing HbA1c, 2H glucose tolerance and fasting glucose), lipid metabolism (reducing Triglyceride, Total and LDL-cholesterol and increasing HDL cholesterol), physical fitness (increase), systolic and diastolic blood pressure, body composition (reducing measures of fatness, body weight, body mass index and increasing lean body mass). Moreover, we expect that the U-TURN intervention is superior in inducing partial T2D remission and reducing body weight with ≥ 5% and ≥10% compared to standard care at time point 24 months. The continuous secondary outcomes will be analyzed using repeated-measures analysis of covariance applied in mixed linear models. Within the framework of repeated-measures linear mixed models, an analysis of covariance model will be used to analyze mean changes in continuous endpoints. The model includes treatment (group), time (3, 6, 9, 12 or 24 months), sex, and the possible interaction between treatment (group) and time (months) as fixed effects, with the baseline value of the

relevant variable as a covariate. Dichotomous endpoints will be analyzed with the use of logistic regression models as described above.

MEASUREMENTS TO ADJUST FOR MULTIPLICITY, CONFOUNDERS, HETEROGENEITY

As the primary outcome of the original study was evaluated at time point 12 months[1], all measures in this U-Turn extension study are considered supportive of the primary findings and are thus meant as exploratory. Thus, we will not adjust for multiple comparisons per default, rather we will interpret the data cautiously taking multiplicity into account.[10]

To explore the influence of heterogeneity in baseline glycemic control and initial medications usage, baseline (time point 0 months) HbA1c, fasting glucose and medications score and sex as co-variates in the models for complete or partial remission include following the crude analysis (with only group and sex as fixed factors).

Subgroup analyses

Planned subgroup analyses will include a comparison of outcome (net benefit) on:

1) patients of different sex (“female” vs. “males”)
2) age; median split (“Young T2D” vs. “Old T2D”). Median split is predefined due to considerations of low statistical power and that no clinically meaningful cut-offs are defined on age and short diabetes duration.
3) impaired glucose tolerance (IGT) defined as 2h glucose during a 2 hour OGTT glucose ≥11.1 mmol/L
4) Impaired fasting glucose (IFG); defined as fasting glucose >=7.0 mmol/L
5) IFG and IGT

6) diabetes duration (time since diagnose at time point 0 months)); Median split (short vs. long duration) at entry to the trial. Median split is predefined due to considerations of low statistical power and that no clinically meaningful cut-offs are defined on age and short diabetes duration.
7) Cardiorespiratory fitness (“low”=lower than the population mean for age and sex or “high” = higher than the population mean for age and sex)[11]
8) the need for glucose lowering medications at baseline (time point 0) (≤1000 mg Metformin =”low” and >1000 mg Metformin =”high”.
9) Obesity at baseline. Obesity is defined as body mass index >30.
10) Per protocol from time point 0 to 12 months*:* The data collection in the *per-protocol population* is independent of group allocation. The measured outcomes at both baseline and after 12 months are available for analysis for both groups (i.e. complete case). We define the *per protocol* population as participants (all criteria present):

    U-TURN (intervention group):

    a. Attending at least four (of five) medical consultations,
    b. Conducting >=70% of all exercise sessions (supervised and un-supervised – as assessed by the exercise registration)
    c. Only gets the prescribed medications and/or the prescribed combination of medications according to the treatment algorithm.

    Standard care (control group):

    a. Attending at least four (of five) medical consultations,
    b. only gets the prescribed medications and/or the prescribed combination of medications according to the treatment algorithm.

Subgroups difference will be reported in strata of the pre-defined groups adapting the 'worst case' scenario approach *a priory*[4]. That is, if no data is available on the primary outcome at time-point 24 months it will be assumed that the participants did not reach remission as described above. Statistical test of interaction will be performed (comparing whether the net benefit of U-turn over comparator varies with subgroup)[12]; data will be presented in "forest plot format".

SENSITIVITY ANALYSIS

To explore sensitivity on the primary outcome, we will impute missing values on the basis of worst case/ best case scenarios. Thus, for participants lost to follow-up at time point 24-month (did not attend the assessment or provide any data) we will impute remission= YES (did obtain complete remission – best-case approach) or remission = NO (did not obtain complete remission – worst-case approach). Patterns of missing data will be investigated. *A priori*, the less restrictive missing at random (MAR) assumption is considered more reasonable than the missing data be missing completely at random (MCAR). Assuming that the data on potential drop-outs are missing at random multiple imputation procedures would be applicable to handle missing data for all participants with baseline measurements.

PROGRAMMING PLAN

The program code for the analysis of the primary outcome in Stata is;

*************************** Primary analysis**************************************

logit *remission group sex*, or

****

The variable *remission* represents partial or complete remission as a dichotomous variable containing whether the participants obtained partial or complete remission at time point 24

months (0= NO remission and 1= YES remission). *group* is the group variable (Standard care = 0 and intervention = 1) and *sex* designates the sex of the participant (female = 0 and male=1).

DEVIATION FROM THE CLINICAL TRIALS REGISTRATION

Some minor deviations from the clinical trial registration have occurred. They include an extension of the adverse events definition. For some outcomes, the trial registration was updated for the observational follow-up. This extension and update were reported to clinicaltrials.gov prior to the initiation of the data collection at time point 24-months.

QUALITY CONTROL PLAN

Implementation of the statistical analysis plan

The SAP will be used as a work chart for the statistical analysis and for drafting and completing the study report (scientific article). The SAP will be implemented using the following steps:

1. A data-collection form will be outlined in collaboration between the data-analyst (Mathias Ried-Larsen) and the statistical advisor (Robin Christensen).
2. All data has been entered twice into the data base and checked. In case of in-consistent data, the data from original participant journal is used in the data collection form.
3. The data-analyst will analyze the primary and secondary outcomes
4. Following the analysis, the data-analyst will designate the groups *treatment 1* or *treatment 2* in the figures and tables. As we have performed a 2:1 randomization, the N will provide information on the allocation and will thus be removed in the tables and figures. Thereby the data analyst will leave the writing committee blinded to the treatment allocation. Moreover, as the writing committee will be aware of the outcomes at time point 0 and 12 months, these

will not be disclosed until consensus on the interpretation of primary outcome for the observational follow-up study has been reached.

5. The results will be presented to the writing committee members (by MR-L), who will provide their blinded interpretations of the primary outcome. Uncertainties will be clarified before breaking the allocation.

The writing committee consists of Mathias Ried-Larsen, PhD, (Principal investigator, data analyst); Mette Y. Johansen, MSc.; Chris MacDonald, MSc.; Katrine B. Hansen, MD, PhD; Robin Christensen, MSc, PhD (Statistical advisor); Anne-Sophie Wedell-Neergaard, MD; Nanna Skytt Pilmark, MD; Henning Langberg, PhD; Allan A. Vaag, MD, PhD; Bente K. Pedersen, MD, PhD, Kristian Karstoft, MD, PhD

Anticipated outline of the study report (manuscript)

Figure 1: Flow diagram of the participants in the different parts of the process in this parallel group randomized controlled trial (including enrolment, allocation, follow-ups at time points 0, 12 and 24 months and analysis)

Table 1: Reporting of the baseline participant characteristics (for primary and secondary outcomes) at time points 0 and 24 months. The characteristics will be presented per group and in total as means (standard deviation) or medians (interquartile range). Dichotomous and categorical data will be presented as actual numbers (%).

Figure 2: T2D remission and its components. Figure 2a; the proportion (% - bar chart) of participants obtaining complete or partial T2D remission at time point 24 months for both treatments (intervention and standard care group) with 95% confidence intervals. Figure 2b; fasting glucose at time-points 0, 12 and 24 months with 95% confidence intervals (Least squares means derived from mixed linear models and adjusted for the baseline value). Figure 2c; HbA1c at time-points 0, 12 and 24 months with 95% confidence intervals (Least squares means derived from mixed linear models and adjusted for the baseline value). Data will be presented *as-observed* – i.e. without imputations.

Table 2 Between-group comparisons of the changes in the on the primary and secondary outcomes from time point 0 to 24 months for the ITT population. The results will be reported as the difference in change between the treatments with 95% confidence intervals as described in the statistical analysis section. Non-normally distributed data will be presented as median change with interquartile range. Categorical secondary endpoints will be reported as proportions (%), odds ratios or relative risk and absolute risk difference with 95% confidence intervals.

Figure 3: Group difference and sub-group analyses for partial or complete T2D remission; Pre-specified sub-group analyses depicted in a forest-plot.

Online only supplementary material

eFigure 1: Least squared means with 95% confidence intervals (per group) for HbA1c at time points 0, 3, 6, 9, 12 and 24 months follow up for the ITT population. Data will be presented *as-observed* – i.e. without imputations.

eFigure 2: Least squared means with 95% confidence intervals (per group) for the body weight at time points 0, 3, 6, 9, 12 and 24 months follow up for the ITT population. Data will be presented *as-observed* – i.e. without imputations.

eFigure 3: Least squared means with 95% confidence intervals (per group) for the lean body mass at time points 0, 3, 6, 9, 12 and 24 months follow up for the ITT population. Data will be presented *as-observed* – i.e. without imputations.

eFigure 4: Least squared means with 95% confidence intervals (per group) for the total body fat percentage at time points 0, 3, 6, 9, 12 and 24 months follow up for the ITT population. Data will be presented *as-observed* – i.e. without imputations.

eTable 1: Odds ratios or risk ratios and risk differences for obtaining complete and partial or complete type 2 diabetes remission (primary endpoint) at time point 24 months with 95% confidence intervals. Missing values are imputed on the basis of worst case/ best case scenarios. Thus, for participants lost to follow-up at time point 24-month (did not attend the assessment or provide any data) we will impute remission= YES (did obtain complete remission - best case approach) or remission = NO (did not obtain complete remission – worst-case approach).

eTable 2: Self-reported physical activity and dietary intake at time-points 0, 12 and 24 months. Data are presented as mean and standard deviation or median and interquartile range. Data will be presented *as-observed*.

eTable 3: Self-reported medicine adherence at time-point 24 months. Data are categorical and are presented as numbers and frequencies for glucose, blood pressure, and lipid-lowering medication, respectively. Data will be presented *as-observed*.

REFERENCES

1. Johansen MY, MacDonald CS, Hansen KB, et al. Effect of an Intensive Lifestyle Intervention on Glycemic Control in Patients With Type 2 Diabetes: A Randomized Clinical Trial. *JAMA : the journal of the American Medical Association.* 2017;318(7):637-646.
2. Buse JB, Caprio S, Cefalu WT, et al. How do we define cure of diabetes? *Diabetes care.* 2009;32(11):2133-2135.
3. Ried-Larsen M, Christensen R, Hansen KB, et al. Head-to-head comparison of intensive lifestyle intervention (U-TURN) versus conventional multifactorial care in patients with type 2 diabetes: protocol and rationale for an assessor-blinded, parallel group and randomised trial. *BMJ open.* 2015;5(12):e009764.
4. White IR, Horton NJ, Carpenter J, Pocock SJ. Strategy for intention to treat analysis in randomised trials with missing outcome data. *BMJ (Clinical research ed.).* 2011;342:d40.
5. Deeks J. When can odds ratios mislead? Odds ratios should be used only in case-control studies and logistic regression analyses. *BMJ (Clinical research ed.).* 1998;317(7166):1155-1156; author reply 1156-1157.
6. Altman DG, Deeks JJ, Sackett DL. Odds ratios should be avoided when events are common. *BMJ (Clinical research ed.).* 1998;317(7168):1318.
7. Karter AJ, Nundy S, Parker MM, Moffet HH, Huang ES. Incidence of remission in adults with type 2 diabetes: the diabetes & aging study. *Diabetes care.* 2014;37(12):3188-3195.
8. Hernan MA, Robins JM. Per-Protocol Analyses of Pragmatic Trials. *The New England journal of medicine.* 2017;377(14):1391-1398.
9. Sterne JA, White IR, Carlin JB, et al. Multiple imputation for missing data in epidemiological and clinical research: potential and pitfalls. *BMJ (Clinical research ed.).* 2009;338:b2393.
10. Bender R, Lange S. Adjusting for multiple testing--when and how? *J Clin Epidemiol.* 2001;54(4):343-349.
11. Shvartz E, Reibold RC. Aerobic fitness norms for males and females aged 6 to 75 years: a review. *Aviation, space, and environmental medicine.* 1990;61(1):3-11.
12. Altman DG, Bland JM. Interaction revisited: the difference between two estimates. *BMJ (Clinical research ed.).* 2003;326(7382):219.
