# Supplementary material for: Type 2 diabetes remission 1 year after an intensive lifestyle intervention: A secondary analysis of a randomized clinical trial
Source: Diabetes Obes Metab. 2019 Jun 30;21(10):2257–66. doi: 10.1111/dom.13802 (PMC6772176; doi:10.1111/dom.13802)
Supplement: Supplementary file 3 — Appendix S2. Statistical analysis plan. [file DOM-21-2257-s003.pdf]

# Feasibility of an intensive lifestyle change in a sample of newly diagnosed patients with Type 2 Diabetes Mellitus (T2DM) – The UTURN project

## A randomized controlled trial

**Registry:** **Clinicaltrials.gov** (NCT02417012)
**Effective date:** **16/08/2016**
**Version:** **3**

## SCIENTIFIC PARTICIPANTS

Mathias Ried-Larsen (Primary Investigator), MSc., Ph.D., Post doc, The Tryg foundation Center for Physical Activity Research (CFAS)
Bente K. Pedersen, MD, DMsc, professor, CFAS
Allan A. Vaag, MD, DMsc, professor, CFAS
Henning Langberg, DMsc, Ph.D., Professor, CopenRehab, CFAS
Katrine B. Hansen, MD, Ph.D. post doc CFAS
Mette Y. Johansen, Cand. Scient,
Chris MacDonald, Cand. Scient,
Robin Christensen, Ph.D., Senior Researcher, Parker Institute, Frederiksberg Hospital
Rasmus Ø.Nielsen, MSc, Ph.D. Database manager, CFAS/University of Aarhus
Morten Zacho, MSc., test leader, CFAS
Louise Seier, MSc., test coordinator, CFAS
Katja Kofoed, MSc., study nurse, CFAS
Mette Svane, study nurse, CFAS
Kristian Karstoft, MD, Ph.D. fellow, CFAS
Poul Jennum, MD, professor Glostrup Hospital
Oluf B. Pedersen, Professor, Metabolomics Genetics, University of Copenhagen
Søren J. Sørensen, Professor, Dept. of Microbiology, University of Copenhagen

## FUNDING SOURCES

The Tryg Foundation

Content

ROLES AND RESPONSABILITIES .......... 2
INTRODUCTION AND RATIONALE .......... 4
RESEARCH HYPOTHESES .......... 11
STUDY OBJECTIVES .......... 11
METHODS .......... 13
PARTICIPANTS, INTERVENTIONS AND OUTCOMES .......... 13
ELIGIBILITY .......... 13
INTERVENTIONS .......... 14
CO-INTERVENTIONS .......... 22
OUTCOMES .......... 26
PARTICIPANT TIME LINE .......... 28
RECRUITMENT .......... 29
ASSIGNMENT OF INTERVENTIONS .......... 34
SEQUENCE GENERATION .......... 34
ALLOCATION CONCEALMENT MECHANISM .......... 34
IMPLEMENTATION .......... 34
BLINDING .......... 34
DATA COLLECTION, MANAGEMENT AND ANALYSIS .......... 36
DATA COLLECTION METHODS .......... 36
RETENTION .......... 41
DATA MANAGEMENT .......... 41
ANALYSIS .......... 43
MONITORING .......... 45
HARMS: .......... 45
ETHICS AND DISSEMINATION .......... 47
RESEARCH ETHICS APPROVAL .......... 47
PROTOCOL AMMENDMENTS .......... 47
CONFIDENTIALTY .......... 48
DECLERATION OF INTEREST AND FUNDING .......... 48
DISSEMINATION POLICY .......... 48
ACCESS TO DATA .......... 49
FEASABILITY .......... 49
REFERENCES .......... 51
APPENDIX .......... 54

## ROLES AND RESPONSABILITIES

**Steering committee:** Design, conceptualizing the trial, reporting of trial results and possible early termination of the trial due to adverse effects will be done by the steering committee. The committee will have no practical role in the data collection, recruitment, randomization and allocation of the patients.

**Coordinating center:** The coordinating of the trial including recruitment and coordination will be done by CFAS (Blegdamsvej 9, 2100 Copenhagen, DK), overseen by PI Mathias Ried-Larsen and Kristian Karstoft. Within the center a separate team will conduct all data collection and a separate team will oversee and manage the intervention including compliance monitoring. Neither team will have a role in the data analysis.

**Data management team:** Data management, generation of the randomization sequence, and allocation will be performed by an independent biostatistician (Prof. Robin Christensen) using an online tool. The data management team will have no roles in the data collection or recruitment of the potential participants.

**Medical regulation team:** The medical regulation team consists of two experienced endocrinologists and will solely assess the need for regulation of the pharmacological treatment of the patients. Dr. Allan A. Vaag will be the primary responsible endocrinologist. The team will have no role in the data collection, data analysis, recruitment, randomization and allocation of the patients.

**Trial sponsor:** Rigshospitalet, Denmark, Blegdamsvej 9, 2100 Copenhagen, Denmark. The sponsor will not have any involvement in conceptualizing the study design; collection, management, analysis, and interpretation of data; writing of the report; and the decision to submit the report for publication

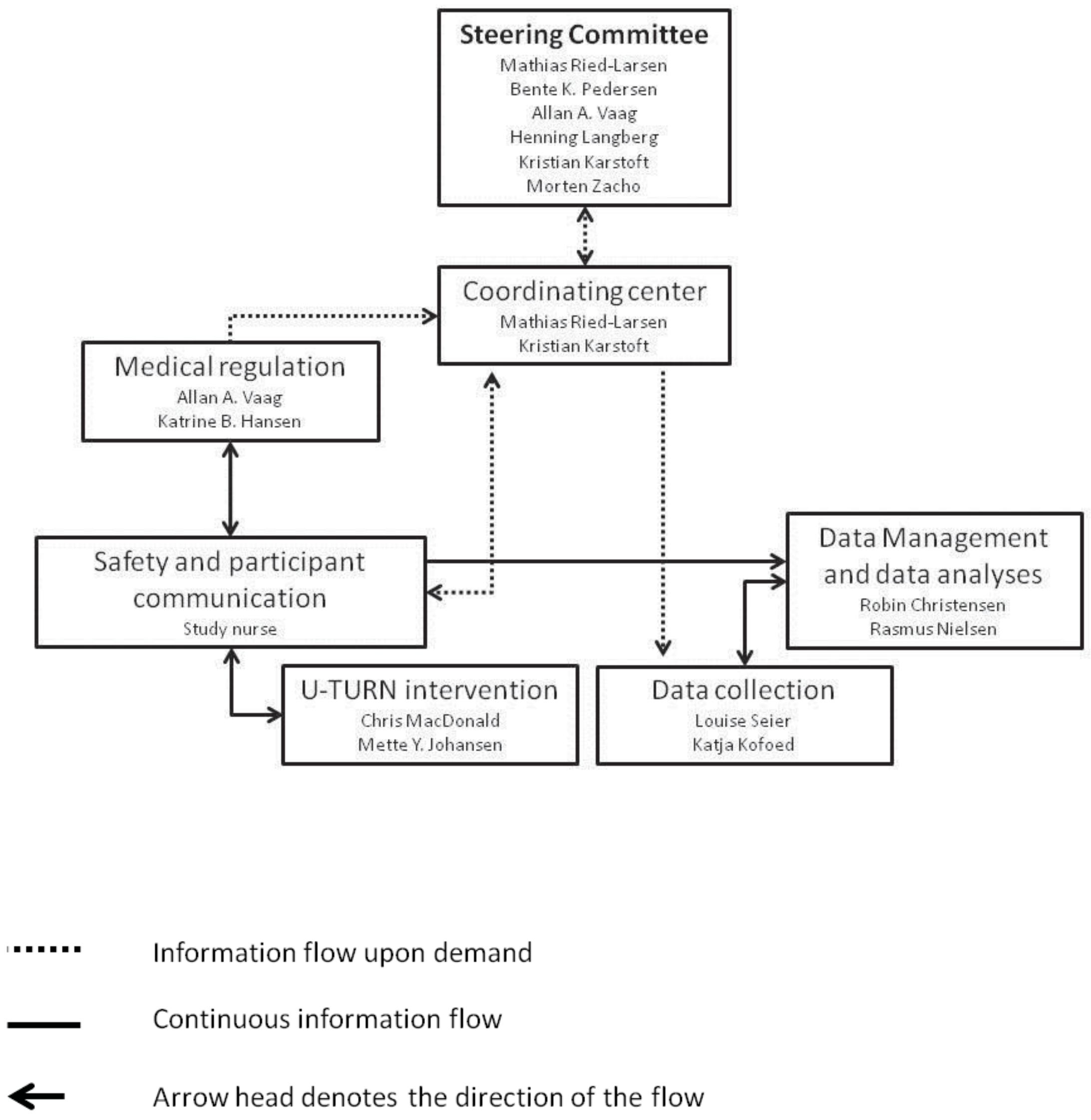


**Figure 1:** The roles of the trial participants, organization of the trial and flow of information.

## INTRODUCTION AND RATIONALE

**The patient population:** The number of patients with type 2 diabetes mellitus (T2DM) is increasing rapidly, with an estimated 439-552 million people diagnosed worldwide by 2030 [1, 2]. T2DM is associated with major personal and economical costs due to an increased prevalence of complications such as cardiovascular disease (CVD), neuropathy, blindness, depression and premature death [3-5]. Although several epidemiological studies have indicated that T2DM is irreversible [6, 7], controversy regarding full or partial remission still exists as several surgical, lifestyle and exercise interventions have shown important clinical improvements in glycaemic control, other cardiovascular disease risk factors and in remission rates [8-11].

**The need for new knowledge:** A recent meta-analysis of 11 randomized controlled trials, multifaceted lifestyle interventions including diet, exercise intervention and combined approaches concluded that lifestyle interventions were only effective in improving glycaemic control when including an intensive pharmacological treatment (Mean difference$_{\text{(pharmacological vs. control)}}$ -0.71 (95%CI -1.31;-0.12) but not without (Mean difference$_{\text{non-pharmacological vs. control)}}$ -0.19 (95%CI -0.46;0.08) [12]. Clearly, intensive glycaemic control using pharmacological approaches are effective in improving glycaemic control in T2DM patients [13]. Thus, when studying the effect of a lifestyle interventions targeting risk behaviour among T2DM patients, the pharmacological treatment needs to be regulated using the same algorithms in the intervention and control groups in order to investigate the effect of the behavioral approach.

In spite of major short-term improvements in weight management and glycaemic control in the largest (N>5,000) lifestyle intervention study (Look AHEAD) study no differences of decreased risk of cardiovascular morbidity, mortality [8] or increased overall partial or full remission between the lifestyle and intervention group were observed [14]. It could be speculated that this could be due to an overall or differential low compliance to the intervention and as

proposed, the increased usage of cholesterol-lowering medication in the control group [8]. Although a recent Cochrane review concluded that the evidence to support that intensive glycaemic control decreased the risk of macro-vascular complications in patients with T2DM [15], targeting glyceamic control might be effective in decreasing the risk of micro-vascular complication. Furthermore the effect of tight glycaemic control might depend on the diabetes severity. In an attempt to interpret the clinical results from the largest randomized controlled trials (RCT) investigating the effects of pharmacological regulation of glycaemic control in T2DM patients, Bianchi and Del Prato described the differential effects of tight glycaemic control on pre-mature death, macro- and micro vascular complications [13]. They proposed that intensifying glycaemic control in newly diagnosed T2DM patients as opposed to patients with progressed T2DM could be beneficial in reducing macro- and micro vascular complications, whereas it had no or adverse effects in patients with severe long standing T2DM [13] – point of view also adopted by the American Diabetes Association (ADA) and the European Study of Diabetes [16]. Along these lines, it was observed that partial remission rates were higher in the patients with short T2DM duration in the Look Ahead trial [14]. Thus, it could be speculated that an intensive lifestyle interventions targeting glycaemic control could prove important in a sub-sample in newly diagnosed T2DM patients. This is supported by findings by Salomon et al, indicating that exercise induced effects on glycaemic control is dependent on pre-training glycaemic control [17].

In summary, well-designed studies on interventions targeting risk behaviors such as exercise and poor diet, in patients with T2DM including better estimates of adherence of the intervention are still needed. Furthermore, to illuminate the effects of the targeted risk-behaviors, the studies need to employ the same pharmacological treatment across the entire sample.

**The Trial interventions:**

**Exercise and physical activity**: Exercise is already established as a cornerstone in diabetes management [18, 19]. Current guidelines recommend that T2DM perform at least 150 minutes of moderate to vigorous intensity aerobic exercise on at least three days during the week and with no more than two consecutive days between each bout. In addition resistance training is advised three times per week at moderate to vigorous intensity [19]. The UTURN exercise intervention is more comprehensive than current exercise guidelines and this extended exercise intervention is partly based on the findings of a meta-analysis [20], where Umpierre et al. examined the association between the effects of structured exercise (aerobic training, resistance training or combined) on haemoglobin $A_{1c}$ ($HbA_{1c}$) levels in T2DM in randomized controlled trials. The analysis of eighteen studies demonstrated that aerobic exercise was associated with an absolute reduction in $HbA_{1c}$ of 0.73% compared with control. Four articles showed that resistance exercise was associated with a decline in $HbA_{1c}$ of 0.57% compared with controls and lastly seven studies with combined aerobic and resistance training was associated with an $HbA_{1c}$ reduction of 0.51% compared with control participants. More importantly results from the meta-analysis showed that more than 150 minutes structured exercise per week is associated with a greater reduction in $HbA_{1c}$ levels corresponding to 0.89% in contrast to 0.36%, when T2DM patients are exposed to 150 minutes or less of structured exercise per week. In addition Umpierre et al. published a more recent review [21], where data from 26 randomised controlled trials with either supervised aerobic, resistance or combined aerobic/resistance training for type 2 diabetics were analysed. They found that exercise volume, more specifically the frequency of aerobic exercise, is highly related to the improvements in glycaemic control in T2DM. Each additional aerobic session may produce an additional reduction of 0.39% in $HbA_{1c}$ level, which underlines the possible benefit of high frequency aerobic training as we aim for in the UTURN exercise intervention. There still remains some uncertainty in relation to

the optimal aerobic exercise intensity span for T2DM. In a meta-analysis [22] it was shown that higher aerobic intensity were related to lower $HbA_{1c}$ levels compared to exercise volume. The target aerobic training intensity span of the UTURN intervention (average 60-88% of $HR_{max}$) is in line with current guidelines [19] with moderate to vigorous intensity. The intensity of the UTURN resistance training will also be in line with current guidelines [19].

The beneficial effects of exercise on T2DM are well documented, but also the relationship between physical inactivity and the development of T2DM as well as deterioration of glycaemic control. In a recent meta-analysis [23] of 10 studies there was a 112% greater relative risk of T2DM associated with a large duration of sedentary behaviour. This risk seems to be independent of exercise-like behaviour i.e. at moderate to vigorous intensity. Sedentary time is also related to all-cause mortality, cardiovascular disease, but with the strongest association to T2DM [24]. Therefore, the UTURN intervention is aiming at minimizing physical inactivity by targeting physical activity via daily steps.

**Sleep**: Sleep has obtained increased attention during the latter years in relation to human health and among others the connection between sleep deprivation and T2DM has been investigated, but the field of research is still relatively novel and unexplored. Yet, a recent review [25] has collected the current knowledge of the possible link between sleep and T2DM. When healthy subjects are exposed to sleep restriction corresponding to 4-5½ hours per night for 5-14 nights, insulin sensitivity reduced from 18 to 24%, indicating a deterioration of glycaemic control. Whether sufficient sleep can improve glucose tolerance has only been investigated in a few studies. In one study where three days of catching up on sleep in-laboratory after chronic intermittent sleep resulted in improved insulin sensitivity after 2-hour glucose tolerance test. In another study fasting and postprandial glucose levels were increased with respectively 8- and 14% after three weeks of sleep

restriction and recurring 28-h days. The review highlights the possible link between different sleep disturbances to abnormal glucose metabolism, but sleep is a novel field of research and randomised controlled trials are difficult to perform; yet some causal effects have been found and comprise the focus on sufficient sleep and regularity in circadian rhythm in the UTURN intervention with the aim to optimize endogenous glycaemic control.

**Diet**: The dietary factors related to management of T2DM have been widely investigated, but some questions remain unanswered when it comes to the most optimal macronutrient distribution. The American Diabetes Association (ADA) is moving towards more individualized goals and with a greater focus on macronutrient quality [26]. Current guidelines from the national Diabetes Association are in line with the Canadian guidelines, where individualization in macronutrient distribution should lie within the range of 45-60E% carbohydrate, 15-20E% protein and 20-35E% fat [27]. The UTURN dietary intervention will be in line with these macronutrient distribution spans and will additionally focus on macronutrient quality in particular glycaemic index/load, fat and non-processed foods. In recent reviews with eleven and twelve randomized controlled trials of > four weeks duration Thomas et al. [28, 29] found that low glycaemic index (GI) or load (GL) diets are related to a reduced $HbA_{1c}$ level compared with high GI or GL diets and without compromising hypoglycaemia events and in a randomized controlled trial from 2012 Jenkins et al. found that low GI foods like legumes were related to improved glycaemic control and reduced cardiovascular risk [30]. Additionally, Ajala et al. found in a review and meta-analysis of 20 randomized controlled trials that low GI diets are effective in T2DM management [31]. In the UTURN dietary intervention emphasis will be on low GI and low GL in shape of non-processed foods. Since T2DM is associated with co-morbidities like cardiovascular disease and due to the general consensus that saturated fat intake is related to cardiovascular disease risk [32] the UTURN intervention aims at

reducing saturated fat intake <7E% as proposed by ADA [26]. In a recent review it was found that both prevention and management of type 2 diabetes are highly related to diets rich in whole grains, fruits, vegetables, nuts and legumes and lower on refined grains, red or processed meat and sugar sweetened beverages [32]. The UTURN dietary intervention will stay in line with current guidelines of macronutrient distribution [27] and, will also aim at providing non-processed foods to the participants with focus on quality of macronutrients.

**Self-monitoring and motivation**: As stated above lifestyle (exercise, physical activity, sleep and diet) is essential in relation to both the prevention and management of T2DM, but in order to explore the true effects of lifestyle one needs to ensure a high level of adherence to the given intervention. Self-regulation theory posits that self-monitoring comes before self-evaluation of progress made towards one's goal and self-reinforcement for the progress made [33] thus, the process of changing habits requires well developed self-regulatory skills. Self-monitoring is central to this process and includes deliberate attention to own actions as well as conditions under which they occur. In a review of 22 studies focusing on self-monitoring of diet, exercise or physical activity Burke et al. found that more frequently self-monitoring was significantly and consistently associated with weight loss. However, due to high variability in measures it was not possible to determine the exact frequency or the optimal way of self-monitoring [34]. In a recent meta-analysis it was furthermore found that motivational interventions could enhance exercise adherence, have long-term effects on exercise behaviour and improve self-efficacy [35]. The UTURN self-monitoring intervention is established in order to optimize and measure adherence to the comprehensive lifestyle intervention. Additionally, motivational and educational strategies are implemented to improve compliance and increased insight to the UTURN intervention and lifestyle changes.

**Sub study; Effect of training on endothelian function in patients with type 2 diabetes assessed by flow mediated dilatation**

In the development of atherosclerosis, a disturbance of endothelian function is considered as a key event. A close correlation of endothelial function in the human coronary and peripheral vasculature has been demonstrated. Although no gold standard for the measurement of endothelial function exists, the measurement of endothelial-dependent flow-mediated dilatation (FMD) in a peripheral artery like the brachial or femoral artery, assessed with Doppler ultrasonography, is the most studied method and shows the most promise for clinical application. The technique provokes the release of nitric oxide, resulting in vasodilation that can be quantitated as an index of vasomotor function. It is a well-tolerated, noninvasive, and low-risk procedure.

Exercise training and physical activity (PA) have substantial vascular and cardiac health benefits, and a metaanalysis have demonstrated a significant, positive relationship between aerobic exercise intensity and FMD.

## RESEARCH HYPOTHESES

The UTURN intervention is feasible and is equivalent in maintaining HbA1c across twelve months of follow-up compared to the control intervention while reducing the need for antidiabetic medications.

## STUDY OBJECTIVES

**Primary objectives:**

To investigate the feasibility of the UTURN intervention in a sample of newly diagnosed T2DM patients.

To determine if the UTURN intervention is equivalent to the control intervention in maintain glycaemic control despite regulation of anti-diabetic, lipid lowering and anti-hypertensive drugs.

**Secondary key objectives:** The key secondary objectives are to determine the effects on the usage of anti-diabetic medication 12 month follow-up.

**Other secondary objectives:** To compare different physical and mental health markers between the UTURN and the control groups in respect to:

- The metabolic profile including fasting blood glucose, 2 hour Oral glucose tolerance test (OGTT) blood glucose, OGTT AUC glucose, fasting and OGTT AUC insulin, C-peptide, low- and high density lipoproteins (LDL and HDL), triglycerides, inflammatory markers and cardiorespiratory fitness
- The differences in body composition including lean body mass, fat mass and fat distribution
- Lipid lowering drugs
- Antihypertensive drugs

- Cognitive functioning
- Depression level, mental- and physical well-being
- Motivation
- Obstructive sleep apnea and sleep quality

**Sub-study objectives and hypotheses:**

The research hypothesis of the sub-study is: The U-turn intervention is superior in improving FMD measurements of endothelian function across twelve months of follow-up compared to the control intervention.

**Trial design:** The UTURN project is a parallel-arm single-blinded randomized controlled trial where the primary end-point is Hba1c monitored across 12 months. Secondary endpoints are monitored until 24 months. The participants are randomized in a 2:1 fashion into an intervention and a control group.

# METHODS

## PARTICIPANTS, INTERVENTIONS AND OUTCOMES

**Study setting:** The intervention is performed in uncontrolled (free-living) environment, whereas all data collection will be performed at the CFAS and Glostrup Hospital. Subjects will be measured at six occasions using a standardized testing battery at baseline, three, six, nine, twelve and 24 months follow-up. Participants are recruited from the Capital Region of Denmark and the nearby surrounding area due to the large number of tests that are located at CFAS.

## ELIGIBILITY

All participants must provide oral and written informed consent before any study procedures are initiated (see appendix for consent form).

***Inclusion Criteria:*** Participants must comply with all of the following at the initial medical examination

1. Less than three anti-diabetic medications.
2. Age >18 years
3. Diabetes 2 duration<10 years
4. BMI >25 but <40 kg/m2,
5. Accept of medical regulation by the UTURN endocrinologists only
6. Accept of purchasing a fitness club membership through UTURN collaborator (www.fitnessdk.dk)

***Exclusion Criteria:*** If the potential participant meets any of the following criteria during the screening procedures, participation will be discontinued:

1. Hba1c> 9% (75 mmol/L)
2. Insulin usage
3. Presence of one or more of the following micro- and macrovascular complications of T2DM;
    a. Diabetic retinopathy (except mild nonproliferative retinopathy or early proliferative retinopathy)
    b. Macro-albuminuria or nephropathy
    c. Diabetic neuropathy (except mild affected vibratory testing (<50 Volt))
    d. Arterial insufficiency
    e. Ischemic heart disease
4. Steroid treatment until three months before the medical examination
5. Thyroid disease (TSH raised/below the normal range)
6. Liver disease (ALAT/ASAT thrice normal range)
7. Inability or contraindication to increased levels of physical activity [36]
8. Evidence of anaemia
9. Lung disease (except mild asthma and mild chronic obstructive pulmonaty disease)
10. Heart disease
11. Kidney disease (creatinine above 130 µM or macro albuminuria)
12. Pregnancy

**INTERVENTIONS**

**Lifestyle intervention:** The UTURN lifestyle intervention will be conducted over a one-year period. Two intervention coordinators (Mette Y. Johansen and Chris MacDonald) will be responsible for the coordination, implementation and compliance of the intervention. In the beginning of the intervention the intervention coordinators and health personnel will to a high

extent provide knowledge and support the implementation and adherence to the intervention. However, it is also our aim to reduce this support towards the end of the intervention in order for the participants more personally responsible.

**Figure 2: Intervention overview**

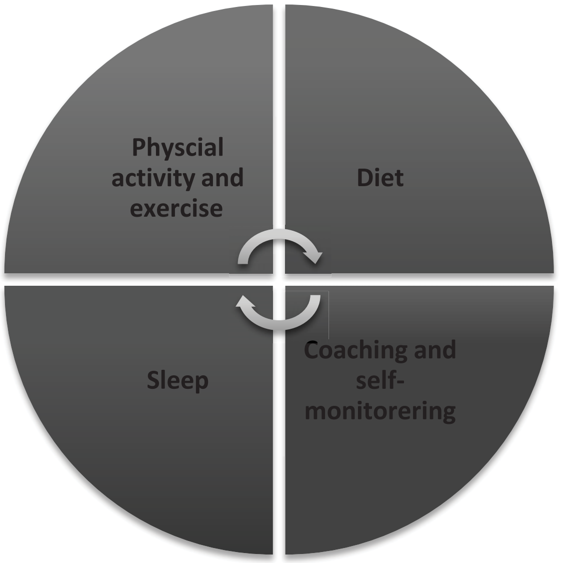


***Physical activity:*** The UTURN lifestyle intervention consists of a physical activity arm: The aim is to reach an individual level of minimum 10.000 steps per day by gradually increasing the number of daily steps within the first month of intervention. Participants are encouraged to use walking as transportation when possible and to take active breaks during the working day to increase daily physical activity. Polar V800 (See description below) will be used to track daily steps and measure compliance. Data must be uploaded to the Polar server on a daily basis or at least every other day.

***Exercise*:** The UTURN lifestyle intervention also consists of an exercise arm with both aerobic and resistance training. After baseline testing and randomization the intervention group is divided into smaller exercise groups consisting of eight participants, 10 groups in total. Each group will be

associated with two certified health personnel, but only one will be present at each training session. The intervention coordinators will provide overall training guidelines on a weekly basis to the health personnel and thereby exercise instructions will be the same across all training groups in relation to the distribution of aerobic and resistance training, including intensity and duration of the aerobic exercise. The type of aerobic training and resistance training way vary between groups. All training will take place in outdoor recreational facilities or in fitness centres. The participants will pay for access to the fitness centres at maximal cost of Dkr. 75/month.

| | Endurance | Resistance | Supervision |
|---|---|---|---|
| Phase 1: 0-4 months | Six times per week<br>30-60 min at<br>62-80 % of HRR (average) | Two times per week<br>30 min | All training |
| Phase 2: 5-9 months | Six times per week<br>45-60 min at<br>68-88 % of HRR (average) | Three times per week<br>30 min | Four weekly<br>trainings |
| Phase 3: 10-12 months | Five times per week<br>60 min at<br>68-88 % of HRR (average) | Three times per week<br>30 min | Two weekly<br>trainings |

Table 1: Endurance training, whole body strength training and supervision of training sessions.

Table 1 illustrates an overview of the advised UTURN exercise intervention. All training will be measured with Polar V800 (Polar Electro, Holte, DK) primarily to monitor aerobic intensity, frequency and duration. Heart rate monitors will also be used to monitor compliance to the resistance-training program, not in relation to intensity, but the ability to complete the advised frequency and duration. On a daily basis or every other day participants have to upload data to the server from the Polar V800 in order for the coordinators to register duration, frequency and intensity of the daily training. In addition the health personnel will report weekly where each

training session has taken place: Indoor or outdoor. When health personnel do not supervise training, participants will have to report to the coordinators where the training has taken place.

***Sleep:*** Regular bedtimes and regular waking times are recommended throughout the week aiming at 7-8 hours of sleep every night, which requires additional 15-20 min in bed in order to fall asleep. All individuals will be recommended to shut down all electronic devices and dim the light at least 30 minutes before bedtime. Participants are requested to use the Polar V800 on a daily basis for monitoring sleep quality and quantity. The sleep data will be uploaded at the same frequency as exercise and will be used to measure compliance.

***Diet:*** The macronutrient distribution in the U-TURN dietary intervention will be in line with current recommendations (45-60E% carbohydrate, 15-20E% protein and 20-35E% fat (<7% saturated fat)), but with individual differences in order to heighten compliance to the dietary intervention. Emphasis will be on low glycaemic index foods and low glycaemic load foods with special emphasis on non-processed foods (e.g. vegetables in particular leafy, fruits and berries, wholegrain and high quality fish, seafood, poultry and meat) and a minimum of processed foods. The diet will be primarily plant based without being vegetarian. The food items will cover on a daily basis three main meals and snacks.

Meal plans will be prepared (see examples for recipes in appendix) by a diet councillor. The meal plans will cover all six meals during the initial four months and supervise the participant’s meal plans during the remainder of the intervention period. Parallel with the exercise intervention the diet councillor will prepare cooking classes and group education (groups will be formed on the basis on the exercise groups – see above) on how to develop a meal plan and implement the plan. In relation to food compliance participants will have to send daily updates on

diet (*see coaching section*), but furthermore participants will perform a dietary frequency questionnaire at baseline and 12 months to quantify compliance in relation to the dietary intervention and to compare macronutrient distribution in the conventional diet with the UTURN dietary intervention.

***Self-monitoring and coaching:*** The UTURN lifestyle intervention will also entail a daily self-monitoring arm, which is based on subjective evaluation. A simple feedback template is made with six questions and a scale from 1 to 10. Each participant has to register and send ranking to one of the intervention coordinators for registration. It is also possible to rate 2, 4, 9, etc. The examples below are just guidelines for the quantification.

- *Exercise:* 1 = I did not train as scheduled, 5 = I completed the training, but with lower intensity than advised, 10 = I completed the advised training.
- *Daily physical activity*: 1 = I walked <2000 steps today, 5 = I walked 6000-7000 steps today, 10 = I walked > 10.000 steps today.
- *Diet*: 1 = I didn´t stick to my dietary plan at all, 5 = 2 out of 3 main meals were in line with my dietary plan, 10 = I followed the dietary plan 100%.
- *Sleep (register from the previous night)*: 1 = I slept < 6 hours. My sleep was very disturbed and I had trouble falling back into sleep, 5 = I slept 7-8 hours. Awake several times, but fell asleep quickly, 10 = I slept 7-8 hours and no major disturbances.
- *Stress*: 1 = A very stressful day, 5 = somewhat stressful, but also peaceful at times, 10 = Balanced stress and I feel good.
- *Mood/motivation*: 1 = I´ve been in a bad mood and my motivation was low, 5 = my mood and motivation could be better, but could also be worse, 10 = my mood and motivation has been at the top.

- *Adherence to the meal plan*: Did well you follow your meal plan? Answer categories 1) "I eat all six meals", 2) "I eat 4-5 of the meals", 3) "I eat 2-3 meals", 4) "I eat <2 meals". I case of 2-4 "Which meals did you skip"?

The self-monitoring serves two primary purposes: 1) To create a heightened awareness in each participant about their daily lifestyle choices in order to increase compliance and 2) To gain insight into each participants' everyday life and overall compliance to the intervention. In addition it offers the opportunity to adjust and individualize programs within the overall frame, e.g. diet by substituting one vegetable with another (more or less same macronutrient content) for higher compliance. Furthermore, the participants are asked to register their diet across 9 days using a costume template for diet registration.

**Administering the self-monitoring and feedback to the participants:** Automated short online questionnaires inquiring on the adherence to the exercise and diet plans, mood/motivation, stress as well as sleep quality will be sent to the participants every morning. The content is briefly described in the intervention description (above). Questionnaires resembling the online questionnaires are found in the appendix. If the adherence is low (reporting <3 in any of the items for >2 days in a week) the intervention coordinators will contact the participants by phone to interview and coach them and help them to increase adherence. All contacts and reasons for contact will be registered in the database. During phase 1 (see table 1) participants receive weekly feedback by mail based on their self-monitoring and objective exercise, sleep and physical activity level measured by Polar V800 both when being compliant, but in particular when being non-compliant as defined above. During phase 2, the feedback will be given every third week and during phase 3 feedback will occur on a monthly basis.

**Participant education and networking:** In addition to the coaching and self-monitoring arm there will be an educational and informative element, where the entire intervention group will participate in 4 x two-hour lectures. This will be established when all participants are recruited and have initiated the intervention in order to gather all participants and speakers at the same time. The focus will be exercise, diet, sleep, stress balance, disease pathology and motivational science in order for the participants to gain insight and knowledge of their disease pathology and the importance of lifestyle in the management of T2DM. Within a week after each session participants will receive a brief summary with key points. These will be generated following the specific events as they will be driven by specific questions from the participants and thus cannot be generated in advance.

After baseline testing and randomization participants will receive a link by mail to a closed web based group on Facebook. In a recent review possible beneficial and disadvantageous are discussed in relation to using social media to amongst others health promotion [37]. It is essential to be aware of privacy settings and thus UTURN participants and health personnel will be requested to keep their engagement in the closed group and not "friend" each other at Facebook. In this closed Facebook group participants are also requested to keep a respectful tone towards each other at all time. During phase 1 (see table 1) the intervention coordinators will post a weekly notice with positive encouragements or encourage to share success, fear, hope etc. and this short of encouragement could potentially create a strong community feeling and enhance the face-to-face interaction [37]. In phase 2 the frequency will be every other week and in phase 3 on a monthly basis. According to the review by George et al. these closed forums are also useful for overall messages to the entire group i.e. in this setup cancelled training or changes in the food items delivered at their doorstep.

**Training of Intervention personnel:** The intervention coordinators will be in charge of the UTURN lifestyle intervention, with assistance and support from certified health personnel. We will recruit 20 health personnel with minimum a bachelor degree in sports science or educated physiotherapists. The primary responsibility and working tasks for the health personnel will be to ensure that as many participants as possible reach the predetermined daily exercise benchmarks (*see table 1*). Health personnel with a high academic and experience level are preferred, due to the fact that they will be responsible for motivating participants and making individual adjustments to the participants' training programs in order to reduce the risk of excessive load or injuries. All health personnel will attend a certification course, which will take place in a weekend prior to the intervention start. It is a requirement that all health personnel attend this certification weekend. During this weekend health personnel will be informed about the following:

- The research protocol
- Disease pathology (type 2 diabetes)
- The intervention: Organizations, exercise, physical activity, diet, sleep and coaching
- Motivation
- Medical issues during intervention (including a course in cardiopulmonary resuscitation)

The intervention coordinators will be in direct contact with the health personnel on a weekly basis. In this way, health personnel will be able to and have to report all issues related to the intervention to the coordinators to take care of and all changes will be made from top to bottom and be streamlined through all groups.

**Control (reference group):** Participants allocated to the control group will follow the Danish standard care. In Denmark newly diagnosed T2DM patients are stratified based on the capability and severity of their condition, primarily by the patients general practitioner (GP), to receive

varying level of rehabilitation and clinical care [38]. However, the pharmacological treatment will be administered solely by the UTURN endocrinologists. See description below.

**CO-INTERVENTIONS**

**Pharmacological regulation**

The endocrinologists, Dr. Allan A. Vaag holds the medical responsibility for the pharmacological treatment. The medical regulation will be performed by Dr. Allan A. Vaag and Dr. Katrine B. Hansen. Due to blinding to group allocation, the study nurse is responsible of the main contact of patients. However, all necessary information, including information about intervention, medical journal and adverse events on the individual participant is available, but blinded to maintain group concealment. If considered necessary, the blinding will be repealed and the participants will be contacted directly by the endocrinologist. This will be decided on a patient-to-patient basis, based on information provided by the study nurse (see later). The participant GP will be informed about the procedure and encouraged to contact the UTURN project nurse in case of questions (see appendix for letter to the GP).

**Standardization of pharmacological treatment:** Following the medical screening all eligable participants will have their anti-diabetic pharmacological treatment standardized using Metformin only potentially supplemented by a GLP-1 analogue. Anti-hypertensive and cholesterol lowering treatment will be standardized using the following pre-specified products:

Biguanid (Tabl. Metformin®), GLP-1 analog (Inj. Victoza®), DPP-4 inhibitor (Tabl. Januvia®), insulin (Inj. Insulatard®), angiotensin receptor antagonist, AT2 (Tabl. Losartan®), thiazide (Tabl. Centyl cum KCL®), calcium antagonist (Tabl. Amlodipin®), a mineralocorticoid (Tabl. Spironolacton®), statin (Tabl. Simvastatin® or Tabl. Atorvastatin®).

**Regulation of medication (before baseline):**

HbA1c, blood pressure and lipids are measured at medical screening. At initiation of the study (after inclusion but before baseline) BT > 150/95 mm Hg, LDL> 2.5 mmol/l or triglycerides > 5 mmol/l or HbA1c > 64 mmol/mol results in initiation or intensification of pharmacological treatment. The UTURN study nurse presents the anonymized data for the blinded endocrinologists, who optimize the medical treatment and if necessary prescribe new additional treatment. The participants needing regulation of medication prior baseline test, are contacted by the study nurse and instructed in optimizing of medication either by phone or – if necessary by an information meeting. The nurse is specialized in diabetes treatment with solid experience from a diabetes out-patient clinic. During UTURN study, she works under responsibility and in close relationship with the UTURN endocrinologists.

**Regulation of medication (baseline to 12 months):** The study nurse presents the anonymized data for the blinded endocrinologists every third month and a decision on regulation of anti-diabetic (baseline and every third month), cholesterol (baseline and every sixth month) and/or antihypertensive medication (baseline and every third month) is made. No information on group allocation is provided to the endocrinologist (Allan A. Vaag and Katrine B. Hansen). The decisions will be based on Hba1c, cholesterol and home blood pressure measurements (eighteen home-based measurements over three days before each test round) using the algorithms described below.

Treatment goals are BP 130/80, LDL/TG <2.0 mM/5 mM or HbA1c < 48mmol/mol. At following controls, BP > 140/95 mm hg, LDL/TG <2.0 mM/5 mM or HbA1c > 58 mmol/l *or* 5mmol/l increment (compared with last control) result in initiation or intensification of pharmacological treatment.

If insulin treatment is initiated, the pharmacological treatment is adjusted on the basis of home glucose monitoring every 2-4 weeks (see below) Also, in case of insulin treatment an information meeting is arranged with the study nurse to educate the participant in glucose monitoring and injection technique.

If the treatment target is reached, the dose of the compound is halved at the following control (three months later). In case of unchanged values or an additional drop the compound is then discontinued.

**Titration of medication:**

*Antidiabetic medication:* Biguanid (Tabl. Metformin®) is initiated at 500 mg once daily up to 1000 mg twice daily. If treatment goal is not reached then a GLP-1 analog (Inj. Victoza®) is added at 1.2 mg. increasing to 1.8 mg daily.  If adverse effects, a DPP-4 inhibitor (Tabl. Januvia®) is used at 100 mg. daily. If treatment goal is not reached, then insulin (Inj. Insulatard®) is added (0.2 units/kg once in the evening). Insulin is titrated based on self-assessed pre-prandial blood glucose measurements in close corporation with the study nurse.

*Antihypertensive medication:* An angiotensin receptor antagonist, AT2 (Tabl. Losartan®) is initiated  at 50 mg daily up to 100 mg daily. If treatment goal is not reached, then a thiazide (Tabl. Centyl cum KCL®) is added at 2.5 mg increasing to 5 mg daily. If treatment goal is not reached, then a calcium antagonist (Tabl. Amlodipin®) is added at 5 mg increasing to 10 mg daily. If adverse effects, a mineralocorticoid (Tabl. Spironolacton®) is used at 25 increasing to 100 mg. daily.

*Lipid lowering medication*: A statin (Tabl. Simvastatin®) is initiated at 40 mg daily. If treatment goal is not reached treatment is replaced by another statin (Tabl. Atorvastatin®) at 10 mg increasing to 40 mg daily.

**Regulation of medication (12 to 24 months):** All pharmacological treatment for the participants (UTURN and control) will performed by their GP.

**Treatment of sleep apnea**: If sleep apnea is detected (Apnea-Hypopnea Index (AHI) > 15/hour) following baseline sleep testing with cardio-respiratory monitoring (CRM) the participant will be offered sleep apnea treatment, but will still be allowed to participate in the UTURN project. Participants that are diagnosed with obstructive sleep apnea at baseline are offered sleep treatment Continuous Positive Airway Pressure (CPAP) and personnel that are not involved in testing or analyzing data from CRM will administer this. In order to minimize the effect of CPAP treatment on retesting results the participants with obstructive sleep apnea are told to pause the CPAP treatment 1-2 days before retesting.

**Safety criteria:** Safety criteria include adverse events, health related outcomes (for instance episodes of angina or signs of atrial fibrillation) and subject-reported hypo-glycaemic episodes (plasma glucose <4mmol/l).Minor hypo-glycaemic episodes are defined as those that can be self-treated; major episodes are defined as plasma glucose <3mmol/l or episodes requiring third-party assistance or medical intervention. In case of adverse effects, medication is changes according to titration described above. In case of hypo-glycaemic episodes anti-diabetic medication is adjusted. Major hypo-glycaemic periods are reported to the study nurse. The hypo-glycaemic events are then presented to the endocrinologist and registered in the database.

## OUTCOMES

The timing of the measurements on a test day is depicted in Table 2. All measurements will be performed at baseline 12-, and 24-month follow-up. Follow-up at 3-, 6- and 9-month consist of a fasting venous blood sample and a measurement of body composition.

**Table 2. Outcomes**

| | Baseline | 3 months | 6 months | 9 months | 12 months | 24 months | (5 years) |
|---|---|---|---|---|---|---|---|
| **Primary outcome** | | | | | | | |
| Hba1c | V | V | V | V | V | V | |
| **Secondary key outcomes** | | | | | | | |
| Metformin | V | V | V | V | V | V | |
| Insulin | V | V | V | V | V | V | |
| GLP-1 agonist (or DPP-4 inh) | V | V | V | V | V | V | |
| Well being | V | | | | V | V | |
| **Other secondary outcomes** | | | | | | | |
| Body composition | V | V | V | V | V | V | |
| BMI | V | V | V | V | V | V | |
| Body mass | V | V | V | V | V | V | |
| Physical fitness | V | | | | V | V | |
| Cortisol | V | V | V | V | V | V | |
| Inflammatory markers | V | V | V | V | V | V | |
| BDI | V | | | | V | V | |
| Motivation | V | V | V | V | V | V | |
| Cognitive function | V | | | | V | V | |
| Oral glucose tolerance test | V | | | | V | V | |
| Cholesterol lowering medication | V | | V | V | V | V | |
| BP lowering medication | V | V | V | v | V | V | |
| Fasting lipids/cholesterol | V | V | V | V | V | V | |
| Sys BP | V | | V | | V | V | |
| Dia BP | V | | V | | V | V | |
| Fasting insulin | V | | | | V | V | |
| Fasting glucose | V | | | | V | V | |
| Obstructive sleep apnea and sleep quality | V | | | | V | | |
| Well-being | V | V | V | | V | V | |
| Flow mediated dilatation | V | | | | V | | |
| Patient activation measures | | | | | V | V | |
| Time preferences | | | | | V | V | |
| Feacal and urine samples | V | V | V | V | V | V | |
| SHRI | | | | | V | V | |

**PARTICIPANT TIME LINE**

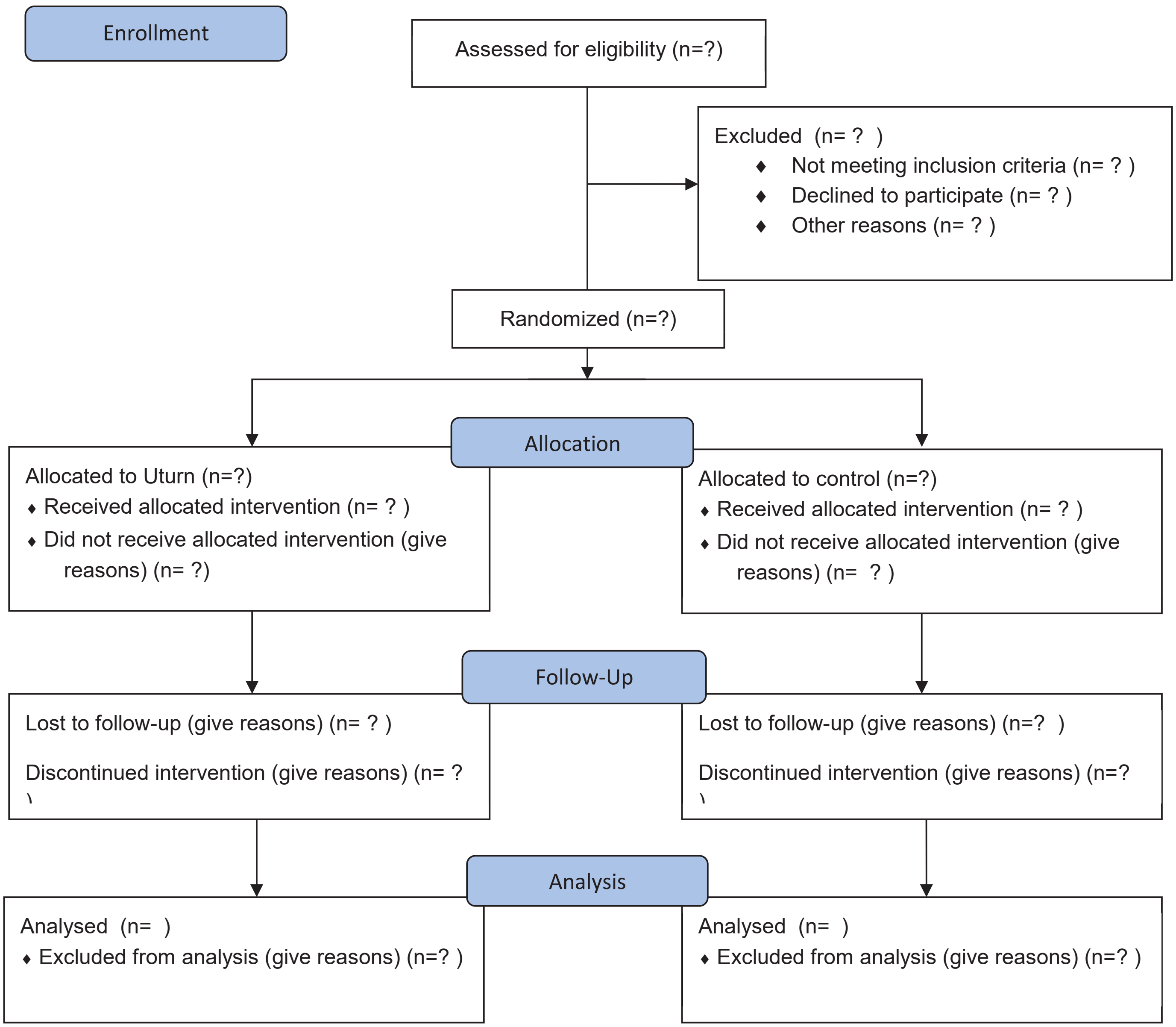


**Figure 3. Flow of participants**

SAMPLE SIZE CONSIDERATIONS

The sample size is this study was based on what was considered feasible, within the local context, enabling up to 120 participants to be enrolled in the trial period. The sample size is truncated at 120 participants or the N reached at the end of recruitment period - whatever is reached first. To increase the sensitivity to the U-TURN intervention it was decided to randomise the participants in a 2:1 fashion.

From the content experts it was decided that a reasonable equivalence margin would ± 0.4%-points for HbA1c for the between group comparison. Assuming that the HbA1c is managed down to 6.5% in both groups, with an SD of 0.9%, we estimated that enrolling 120 participants in the intention-to-treat population (ITT; 80:40), testing a 2-tailed superiority hypothesis (based on 95% confidence intervals [39] would be reasonably precise to estimate within a reasonable equivalence margin; -0.34% to 0.34%). Further, according to the principle of sensitivity, our estimates support that even if we include only 90 (60:30) participants our confidence limits will be acceptable.

**Substudy:** We expect equal variance in FMD in the range (standard deviation (SD)) of absolute change in FMD of 1.5% to 2%. Thus, we employed a SD 2% for the power calculations. A total of 20 patients from the intervention group and 20 patients from the control group participate in the substudy with a statistical power ($1-\beta$) of 0.80 and at a statistical inference level of $\alpha=0.05$.

RECRUITMENT

Recruitment is initiated upon approval from the Regional scientific committee. The procedure is done in three steps. The process is depicted in Figure 4 and the steps are described below.

**Step 1:** The intervention will be announced through social media, press and relevant websites using an advertisement (see appendix) and a press release informing the press about the project. Alongside the recruitment a TV-production team is casting six persons for a TV-show about a

similar lifestyle intervention. This TV-show is NOT a part of the research project. As we are expecting a large amount of queries for only six roles in the TV production, we will mention the study and provide a link to the study advertisement (at www.aktivsundhed.dk) in the refusal letter to the unsuccessful TV-candidates. In the way we will not provide unsolicited study material as the interested persons are actively activating the link.

**Initial screening procedures:** The potential participants will contact the scientific staff (study nurses) by email and provide contact information. Upon contact, the written study information material is send to the potential participant. The person will be encouraged to read the information thoroughly and is informed about the study and the possibility of inviting a private counselor to an information meeting. The participant will be given at least two days to read the material before contact is made. Then the potential participants are contacted by phone and informed orally about the study. During the oral information it will be made explicit that the potential participant is eligible for an information meeting with the study nurse. During the phone information meeting the participants are also informed that they are eligible for time (at least 24 hours) to consider participation before consenting orally and in writing. After the oral information has been provided and if the potential participant is still interested in participation the person is then screened for inclusion and exclusion criteria. The information in this phone screening is **NOT** used for later evaluation of the project. In relation to the phone screening, the study nurse can consult a medical doctor if needed. If the person does not meet the inclusion criteria or any exclusion criteria are identified, the persons are informed hereof and the process is discontinued.

In case of inquiry of an information meeting, the study information will be given by the study nurse in a closed room where only the scientific participant, the potential participant and potentially the private counselor will be present. In relation to the information meeting it is possible for the study nurse to contact one of the UTURN medical doctors if needed. The study nurse

conducting the information will have extensive experience in handling the clinical treatment of T2DM patients. If the potential participant wants additional time to consider whether or not to participate in the study they can return the consent form in the free post envelope (after 24 hours) or an agreement about a phone meeting within r 48 hours is made. The potential participant will be informed of the possibility of contacting one of the scientific participants by phone, in case of questions.

If the interested persons are eligible for participation and oral and written informed consent is provided in this first step of the recruitment procedure, the participant is included in the study and invited to step 2 (see below). The participant is informed that the later steps can result in exclusion if any exclusion criteria are identified. In case of exclusion the participant will be informed about the reason.

As we are expecting a considerable amount of contacts by potential participants (based on previous experiences we expect ~1000 persons), an initial screening by phone is necessary in order to avoid an extensive amount of later screening failures. Screening failures after this initial point will result in an extensive amount of unnecessary blood samples and is extremely time consuming for the interested persons meeting the exclusion criteria at the later stages of the recruitment process. Thus, to avoid putting unnecessary strain on the interested persons and the project economy an initial phone screening procedure is of utmost importance.

**Step 2:** At this step the participants are invited to Rigshospitalet (Biochemical dept. 3011) for an initial blood test. The blood test is performed to identify potential exclusion criteria not identified at step 1. Theoretically, this blood screen can also reveal unknown disease (e.g. kidney or liver disease). In this case the potential participant will be excluded and referred to their GP. If no additional exclusion criteria are identified, the potential participant is invited to Step 3 - a medical screening.

**Step 3:** The medical screening consists of a medical interview (based on a screening form (appendix)), an electro cardiac gram (ECG) and a physical examination including a blood pressure measurement, cardiopulmonary stethoscopy and a foot examination. If no exclusion criteria are identified the participant will be invited to participate in the U-turn study. If blood pressure measurement at medical screening indicate hypertension, the participants are instructed in home pressure measurements. If participants meet criteria of optimizing medication (see "Standardization of pharmacological treatment above") a six week titration period is thus initiated prior baseline testing.

**Substudy (Effect of training on endothelian function in patients with type 2 diabetes assessed by flow mediated dilatation)**After medical screening (step 3 in the initial recruitment) a substudy of 40 participants (20 from the intervention group and 20 from the control group) are randomly picked among eligible participants who gave written informed consent. The procedure is done by the data manager. All personnel involved in the data collection and the PI is blinded to the allocation.

Prior to baseline testing all selected participants is contacted by phone and invited to participation in the substudy. If interested, the written substudy information material is send to the potential participant. The person will be encouraged to read the information thoroughly and is informed about the substudy and the possibility of inviting a private counselor to an information meeting. The participant will be given at least three two days to read the material before contact is made. Then the potential participants are contacted by phone by and informed orally about the substudy. During the oral information it will be made explicit that the potential participant is eligible for an information meeting with the study nurse. During the phone information meeting the participants are also informed that they are eligible for time (at least 24 hours) to consider participation before

consenting orally and in writing. If still interested the participants sign the consenting and send it by mail, so that informed consent is done before the  test day.

In case of inquiry of an information meeting, the study information will be given by the study nurse in a closed room where only the scientific participant, the potential participant and potentially the private counselor will be present.

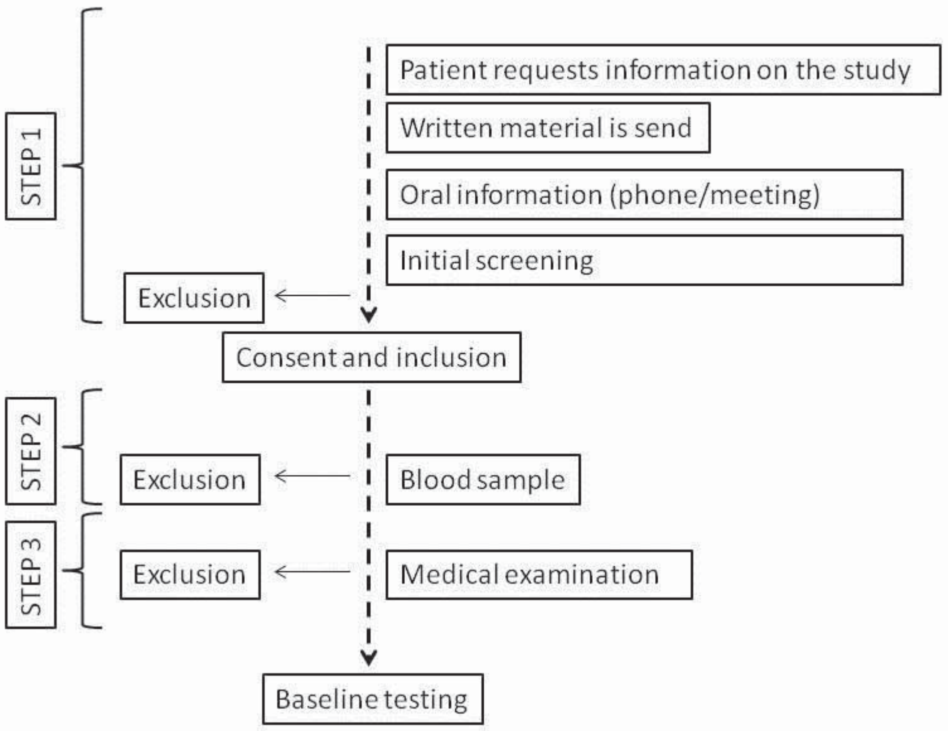


**Figure 4. Assessment of eligability**

## ASSIGNMENT OF INTERVENTIONS

### SEQUENCE GENERATION

Eligible subjects will be randomly assigned to an intervention (UTURN) or a control condition (standard care) after the baseline measurement. Patients are randomized (2:1) using random permuted blocks stratified by sex and geographical location. The unequal randomization is chosen due to financial constraints and constraints in test facilities. The loss of statistical power is considered modest (~5%) when employing a 2:1 randomization [40]. The block sizes will remain unknown for the researchers in order to ensure concealment.

### ALLOCATION CONCEALMENT MECHANISM

Eligible participants will be randomized using the online tool Help2run (see description below), which is an online service administered by the database manager. The sequence is generated and implemented in the web service by the third party statistician. Allocation is not revealed to the test subjects until completion of the baseline measurements and will thus be ensured.

### IMPLEMENTATION

The eligible participants who gave written informed consent are informed about the allocation directly by email age generated by the online tool. The procedure is supervised by the data manager. At the same time the intervention coordinators will receive notice on the allocation. The study intervention coordinators will then contact the participants and initiate either the UTURN or control intervention. The allocation is concealed from the researchers until post 12-month follow-up.

### BLINDING

All personnel involved in the data collection and the PI is blinded to the allocation. The test subjects will be informed that they are not allowed to discuss their allocation during the follow-up measurements. Due to the nature of the trial, participants, project nurse and intervention coordinators cannot be blinded to the allocation.

The data collection personnel will feed the test data into an online database. The test will not be accessible to the researchers until completion of the 12-month follow-up data collection. The data will be completely anonymized before analysis. The analysis plan is developed prior to the intervention and all statistical analyses are performed by to separate researchers (The PI and Biostatistician).

# DATA COLLECTION, MANAGEMENT AND ANALYSIS

## DATA COLLECTION METHODS

**Training of data collection personal:** All personnel will undergo extensive training before baseline testing. A part of the training will include extensive piloting of the test procedure.

**Glucose Tolerance and blood sampling:** Following an overnight fast (8 hours), an antecubital intravenous (i.v.) line will be placed and a standard 75 g oral glucose tolerance test (OGTT) will be performed over 2 hours with blood draws at time 0 (baseline), 15, 30, 60 and 120 min. The blood markers of metabolism (plasma- LDL, HDL, TG, TC, fasting insulin, C-peptide and glucose), inflammation will be analysed. A saliva sample will be collected for analyses of cortisol .These parameters will be assessed at baseline and at 3, 6, 9, 12 - and 24 month follow-up.

**Estimation of $VO_{2max}$:** $VO_{2max}$ will be assessed by employing a progressive bicycle ergometer test protocol. After a 5-min warm-up, the work load is increase every minute. The test will be continued until the following criteria were met: plateauing of heart rate and $VO_2$ with incremental workloads, respiratory exchange ratio > 1.1 [41]. Oxygen consumption will be assessed using continuous indirect calorimetric measurements (CPET, Cosmed, Italy) and heart rate monitoring (Polar Electro, Holte, DK).

**Cognitive testing:** Specific cognitive function areas (short-term memory, attention, executive functions etc.) will be tested using the CANTAB test package from Cambridge Cognition. The tests are conducted on a PC with a touch screen. A comprehensive test takes approx. 30 minutes and consists of 3-6 individual tests, each of which takes 2-10 minutes.

**Beck Depression Index:** The Beck Depression Inventory (BDI) is a 21-item, self-report rating inventory that measures characteristic attitudes and symptoms of depression both physiological and cognitive [42]. The higher score, the higher severity of depression.

**Well-being and functional ability:** The SF-36 is a multi-purpose, short-form health survey with only 36 questions. It yields an 8-scale profile of functional health and well-being scores as well as psychometrically-based physical and mental health summary measures and a preference-based health utility index [43]. The Global Mood Scale measures positive and negative effect on separate sub-scales and comprises 20 questions in total. The scale has been validated with both patients with chronic illness and adults in a working population. The Mental Health Continuum Short Form measures different dimensions of positive mental health and include emotional, psychological and social well-being. It consists of 14 questions distributed on the dimensions of well-being mentioned above. Individuals with high scores on positive mental health can be characterized as Flourishing. The Warwick-Edinburgh Mental Well-being Scale measures subjective well-being and psychological functioning with a focus on addressing positive aspects of mental health. It comprises 14 questions which are all worded in positive terms.

**Motivation:** The Behavioral Regulation In Exercise Questionnaire (BREQ) and its subsequent modification (BREQ-2) have become the most widely used measures of the continuum of behavioral regulation in exercise psychology research.

**Personality traits:** The patient will be asked to complete two questionnaires regarding personality, the NEO-Five Factor Inventory (NEO-FFI) and a Sensation Seeking Scale (SES). The NEO-FFI consists of 60 questions in a 0-4 Likert format constructed by selecting 2 items from each of the six facets characterizing each of the five personality traits (Neuroticism, Extraversion, Openness, Agreeableness, Conscientiousness) assessed by NEO-PI-R [44].

The Sensation Seeking Scale is a 40 items self-administered questionnaire and consists of 40 questions designed to test the tendency towards varied, novel and intense sensations [45].

**Food frequency questionnaire:** The participant will be asked to fill out a food frequency questionnaire at baseline and 12 months. The questionnaire has earlier been used in the national

report: “KRAM - kost, rygning, alkohol og motion”. The questionnaire is sub-divided into food items and the participants have to recall the frequency of the given foods within the last year. Furthermore, the participants are asked to register their diet across 3x3 days. The participants are asked to weight their food.

**Additional samples:**

The patients were asked to collect feacal and urine samples for microbiome analysis. Prior to the collection they were informed orally and in writing about the procedures (according to the procedures about informed consent, as described in this protocol). Bacterial and virome DNA will be extracted and used for characterization of the micro biome in the gut (i.e. no human DNA will be analyzed).

**Demographic Information:** Height, weight, waist and hip circumference will be measured by standard procedures. Dual x-ray absorptiometry (iDXA; Lunar, Madison, WI) and COREScan will be used to assess whole body adiposity. Sociodemographic information and information on the physical activity level will be obtained using questionnaires (Uddannelses- og helbredsspørgeskema/Recent Physical Activity Questionnaire (RPAQ)). Resting brachial BP is assessed in supine position, as the mean of the consecutive three measurements. At every test round person will be asked about recent use of antibiotics.

**Cardiorespiratory monitoring (CRM):** Cardiorespiratory monitoring consists of electromyography of the tibialis anterior muscles, and electrocardiogram including channels of digital oximetry, nasal pressure and respiratory movements (Xtrace thoracic and abdominal straps). CRM is used to measure the prevalence of obstructive sleep apnea.

**Sleep quality and sleep disturbances:** The Pittsburgh Sleep Quality Index (PSQI) measures self-reported sleep quality and disturbances during the previous four weeks. PSQI has 19 items and

measures 7 components of sleep: subjective sleep quality, sleep latency, sleep duration, sleep disturbance, use of sleeping medication, habitual sleep efficiency and daytime dysfunction.

**Sleepiness:** The Epworth Sleepiness Scale (ESS) is a self-reported questionnaire that measures daytime sleepiness. The questionnaire consists of 8 items with a respondents format 0 = would never doze, 1 = slight chance of dozing, 2 = moderate chance of dozing, and 3 = high chance of dozing.

**Sleep pattern:** Patients complete sleep diaries for 14 days after each CRM measurement in order to monitor and describe potential changes in their sleep. The sleep diary contains self-reported information about what time they go to bed and get up, as well as the number of awakenings during the night and daytime dysfunction.

**Fatigue:** Multidimensional Fatigue Inventory (MFI-20) is a self-reported questionnaire with 20 questions to measure fatigue. It covers the following dimensions: General fatigue, physical fatigue, reduced activity, reduced motivation and mental fatigue.

**Compliance:** The Polar V800 (Polar Electro, Holte, DK) will be used to monitor compliance to physical activity (steps) and sleep duration via the integrated accelerometer in the Polar watch. The Polar connector unit which is placed around the chest measures the electrocardiogram (ECG) signal. It detects the QRS complexes with a 1 ms resolution and is used for measuring heart rate during exercise. Heart rate variability (HRV) is measured with Polar V800. The Polar V800 and HR band monitors heart rate on the basis of R-R intervals and this measurement has been validated earlier [White paper: R-R Interval Measurement and Heart Rate Variability in Polar Products, Kaisu Martinmäki & Hannu Kinnunen, May 2011] [46]. Each participant is requested to measure HRV on a weekly basis and these data have to be uploaded for the Polar database. In addition the Polar V800 registers time and frequency of both aerobic and strength training. The dietary compliance will be

based on daily self-monitoring, but in order to quantify compliance baseline and 12 months dietary electronic food frequency questionnaires will be performed to quantify energy intake and macronutrient distribution.

**Patient activation measures and time-preferences:** Patient activation measures will be assessed using the Danish translation of PAM13 [47]. It is a 13 item questionnaire used for characterization of the patient's engagement and knowledge about their health. Time-preferences are determined using a 4-item questionnaire. Both questionnaires will be used to assess the risk of relapse following the termination of the U-TURN intervention.

**Self-Report Habit Index (SRHI):** The Self-Report Habit Index measures habitual behaviour with three open-ended questions and four statements answered on a 5-item scale ranging from "Highly disagree" to "Highly agree". SRHI describes frequency and automaticity of behaviour as well as relevance to self-identity [48].

**Biological material:** Blood will be collected in the study and stored in a research biobank throughout the study. This will be used to assess the potential differential changes in risk variables following the interventions. Approx. 100 mL (at 0, 12 and 24 month), 30 mL (at 3, 6 and 9 months) and 10 ml (medical screening) blood will be collected per trial day. Total blood sampling will be ~400 mL across 24 months. After the study termination the remaining biological material will anonymously get stored in a biobank for a maximum of 20 years and after that, it will be destroyed. If any later studies want to make use of the biological material or some analyses are performed outside Denmark, this will only take place following approval by the ethical committee.

**Substudy, Flow mediated Dilatation**: After 12 hours fasting (including caffeine and tobacco and all vasoactive medications (withheld for at least four half-lives)) the patients are positioned supine with the leg in a comfortable position for imaging the femoral artery.

An ultrasound system equipped with vascular software for two-dimensional imaging, colour and spectral Doppler, an internal electrocardiogram (ECG) monitor and a high-frequency vascular transducer is used for examination. To create a flow stimulus in the femoral artery, a sphygmomanometric (blood pressure) cuff is first placed either on the tight. A baseline rest image is acquired, and blood flow is estimated by time-averaging the pulsed Doppler velocity signal obtained from a midartery sample volume. Thereafter, arterial occlusion is created by cuff inflation to suprasystolic pressure. Typically, the cuff is inflated to at least 50 mm Hg above systolic pressure to occlude arterial inflow for a standardized length of time.

**RETENTION**

All participants will receive DKr. 2,250 to cover lost earnings, transport and discomfort. The transaction is completed upon completion of the study (all lab days or upon withdrawal). For every completed full day of laboratory testing, participants will receive DKr. 750 (at 0, 12 and 24 month follow-up). To ensure a low drop-out in the control, they receive the pharmacological treatment and is invited to a blood sampling a CFAS every third month (the first year). All participants are allowed to contact the UTURN study nurse by phone in case of project related questions (e.g. pharmacological treatment, sports injuries etc.).

**DATA MANAGEMENT**

**Data forms and data entry:** An online-based database is developed to logistically handle the data gathered at baseline and during follow-up. The database is developed by the Danish Stakeholder Help2Run (CVR: 34801088), who also supports the researchers during the project. Data from the data collection procedures at CFAS will be entered directly into the system on-site. Separate data forms will be used for the medical screening, test procedures and questionnaires. The integrity of the data will be secured through double entry of the data in separate forms and data valid rules (pre-

defined ranges, no. digits etc.). During post processing consistency check will be performed by the data managers. Original data files (from the Cosmed and DXA scans), will be stored securely on the hospital server. Compliance data (from the Polar V800) will be send online to the Polar servers, anonymized and retrieved by the test personal and entered into the database.. Biochemical reports are online on the hospital serves. The reports will be printed and the data will be entered manually into the data sheets.

**Homepage:** Data is entered in a database available via a project-specific homepage (URL is to be created)

**Backend system:** A protected back-end system (http://www.runsafe.dk/mtd/admin/) is used to logistically handle the information from each participant in the trial. The system is protected in the sense that access to personalized data from the UTURN participants is only possible using a personalized username and password.

**Access to data in the back-end:** Mathias Ried-Larsen and Rasmus Nielsen are responsible for administering the permissions to the back-end system. Each researcher with access to the backend has a unique username and password for the back-end system. The password is personal and not available to others. It is strictly prohibited to distribute the username and password to others including other researchers in the UTURN project.

**Logging:** All activities in the back-end system are logged in accordance with the rules and regulations from the Danish Data Protection Agency.

**Data download:** It is possible to download an excel sheet with data (demographic characteristics, responses to questionnaires etc) from the back-end system. In case an Excel sheets is downloaded, a computer linked to a university- or university hospital network must be used to ensure that the computer-specific handling of the excel sheet is logged via the university or at the university

hospital. It is prohibited to download data containing personal information of the UTURN participants to a private computer.

**Data sharing:** via e-mail amongst persons outside the study group is performed in accordance with the rules and regulation at University of Copenhagen and Aarhus University. This includes encryption of ID numbers and deletion of personal information.

**Data storage:** Data is stored on two servers. The first server is hosted by Amazon.com with the European servers located in the Republic of Ireland. Back-up of data available on this server is performed on a daily basis. In addition, a server is located at Terndrup Møllevej 95, 8543 Hornslet, Denmark (at Help2Run) to secure access to data in the case the services provided by Amazon.com is unavailable. Back-up on the latter is performed once weekly.

**Security and back up of data in paper form:** All paper forms including consent forms, biochemical reports, ECG prints will be journalized by the test personal and kept in locked cabinets. The entry after the forms are journalized is restricted to the hospital administration (Inge Holm).

**ANALYSIS**

All analyses will be conducted according to the Intention-to-treat principle. Mixed model analyses will be employed to determine the group X treatment effects of the intervention across twelve and 24 months. The secondary outcomes will be analyzed using the same approach. Assuming that the data on potential drop-outs are missing at random, multiple imputation procedures will employed to handle missing data. Patterns of missing data will be investigated. Per protocol analyses of the UTURN group will be performed to support the primary analysis. Explorative analyses of the treatment effects will be performed after 24 months of follow-up and include stratified analysis based on personality types, motivation, and well being. The analyses will be performed

independently by the biostatistician and the PI and results will be compared. In case of discrepancies a third party biostatistician is consulted.

# MONITORING

**HARMS:**

***$VO_2$-max test:*** A physical fitness test, where subjects must put in maximum effort. This will cause some degree of breathlessness. $VO_2$max test is a standard method used for scientific purposes in our laboratory.

***DXA scan:*** Is not expected to cause significant discomfort. The radiation acquired is so small (0.0004 mSv) that it does not pose any risk to subjects. The dose is smaller than received when flying in a commercial jet from (11-12 hours) (SST.dk - Strålingsguiden). DXA-scan is a standard method used for scientific purposes in our laboratory.

**Cardiorespiratory monitoring**: The test is harmless. The electrodes and the equipment can bother the skin and the range of movement, but otherwise no side effects.

***Blood sampling:*** Will cause minor discomfort in terms of a venous catheter. There is theoretically a risk for infections introduced through the catheter. The blood volume collected (miximum 390 ml/24 months is considered so small to cause any symptoms. Blood sampling is a standard method used for scientific purposes in our laboratory. However, there is a small risk of local infection and edemas (phlebitis).

***Substudy: Flow mediated dilatation:*** Will cause minor discomfort due to cuff inflation. The ultra sound examination is harmless.

***Medical regulation:*** Regulation of medication will be performed by an experienced endocrinologist in accordance with the above mentioned pre-defined algorithms. Subjects will be informed about

side effects as well as subjective signs of increased hypo- and hyperglycemia (thirst, polyuria, fatigue, confusion) and urged to contact the study nurse in case of any adverse symptoms.

**Auditing:**

Severe hypo-glycaemic events will be registered by study nurse and the endocrinologist. Furthermore, injuries related to the intervention (acute and over-use) will be registered if reported. In case of reports of severe adverse events during the study period, the steering committee will be informed as will the Scientific Ethical Committee of the Capital Region of Denmark.

## ETHICS AND DISSEMINATION

### RESEARCH ETHICS APPROVAL

The project was initiated by Chris MacDonald, Mette Y. Johansen, Mathias Ried-Larsen, Kristian Karstoft and Bente K. Pedersen. The project is expected to result in limited risks, adverse effects and discomfort to the subjects. The participants can discontinue participation all times with no obligation to provide a reason. The UTURN participants will benefit from the study in terms of a thorough medical examination, increased physical capacity, improved diet and sleep quality and a decreased need of medication. The study is sound and important, and it will contribute to our knowledge about how and if T2DM can be partially remised. The control group will be evaluated supervised continuously by trained endocrinologists, thus assumable improving the pharmacological treatment and general health. Ethical approval will be applied at the Scientific Ethical Committee at the Capital Region of Denmark. Furthermore, the study will be reported to "Datatilsynet" through Rigshospitalets joint review. The "Lov om behandling af personoplysninger" will be respected. The study will be conducted according to the principles of the Helsinki Declaration II.

### PROTOCOL AMMENDMENTS

The substudy, Effect of training on endothelian function in patients with type 2 diabetes assessed by flow mediated dilatation, was initiated by Katrine Bagge Hansen, MD phD and Kristian Karstoft, MD phD. Ethical approval will be applied at the Scientific Ethical Committee at the Capital Region of Denmark as an amendment of the accepted protocol (Protocol ID: H-1-2014-114). Scientific rationale and evidence of potential harms of the content of the intervention or data collection methods can induce changes to the protocol. All potential amendments have to be presented to the UTURN scientific committee before submission to the Scientific Ethical committee of the Capitol Region of Denmark

**CONFIDENTIALTY**

A unique subject ID number will be ascribed to all participants to anonymize data. Identification key (ID to personal information) will be encrypted and stored securely and separately from the unique ID number and database by the data manager.

**DECLERATION OF INTEREST AND FUNDING**

The research project is supported by the TRYG foundation in two grants (Dkr. 2.916M and Dkr. 3M) and by CFAS (Dkr. 2.256M). The total budget for the project amounts to Dkr. 8.172M (See appendix). The first grant and CFAS covers the financial support covers test operating expenses, running expenses for monitoring of adherence, data management and PhD fellow, the salary for test personal, and the compensation to test subjects. This grant is deposited on Rigshospitalets bank account available to the primary investigator (Mathias Ried-Larsen). The latter grant (Dkr. 3M) is granted to the TV collaborator (Ulrik Skotte, DocEye productions) and covers expenses regarding implementation of the intervention. This grant is deposited on a separate account with DocEye productions. All expenses will be documented and signed off by an authorized accountant. The researchers declare no conflicts of interest. The budget is specified in the appendix. If further funding is obtained, VEK is informed and an updated written participant material is send to the participants.

**DISSEMINATION POLICY**

The data will be published in international peer-reviewed journals. The results is to be reported according to the CONSORT guidelines [49]. Furthermore, the findings of the intervention are broadcasted in national Danish television after the twelve months follow-up. All results will be disseminated (negative, positive and inconclusive findings). All results will be published (positive, negative and inconclusive). After the study (after 12 month follow-up) we intend to link study data

with data from the TV-show in order to communicate the experiences with linkage of research and TV-production in the relevant scientific communities.

Topics and ideas for publication (journal papers, abstracts) are to be presented in a written proposal to the UTURN steering committee, including a full list authors. All proposals are sent to the principal investigator (Mathias Ried-Larsen) who will distribute the proposal among the members of the steering committee. The principal investigator or other senior members of the steering committee are considered lead authors of the material derived from the UTURN project. All authors must comply with the "Uniform Requirements for Manuscripts Submitted to Biomedical Journals" [50].

**ACCESS TO DATA**

All data is the property of CFAS. Access to data is overseen by the UTURN steering committee. All UTURN study group members will be granted access to the anonymized cleaned dataset upon completion of the 12-month follow-up data collection and approval of project proposal. The data manager will provide the datasets to the study group members upon request from the steering committee.

**FEASABILITY**

The scientific participants (Post doc Mathias Ried-Larsen (PI), Professor Bente Klarlund Pedersen, Professor Allan Vaag, Professor Henning Langberg, Professor Robin Christensen, Post doc Kristian Karstoft, Post Doc Katrine Bagge Hansen, Cand.Scient Mette Yun Johansen, Cand.Scient Chris MacDonald, Test leader Morten Zacho and Test Coordinator Louise Seier) at CFAS have a track record of conducting and designing interventions studies where the described methods have been employed. Several of the scientific participants have conducted intensive lifestyle and clinical interventions [41, 51, 52] and large scale studies [53, 54] and have great experience in project management and governance.

Due to the unique setup and expectation of clinical significant outcomes we expect that the project can influence the clinical practice and qualify the lifestyle arm of the clinical guidelines. Experiences from the project can inform the therapists in rehabilitation on the practice induce remission through lifestyle change. As the project will be broad casted on national TV we hope and believe that UTURN can inspire T2DM patients to engage in extensive lifestyle change. This can potentially decrease morbidities and mortality and increase quality of life not only in the participants of this project, but also nationally.

# REFERENCES


1. Shaw, J.E., R.A. Sicree, and P.Z. Zimmet, *Global estimates of the prevalence of diabetes for 2010 and 2030.* Diabetes Res Clin Pract, 2010. **87**(1): p. 4-14.
2. Whiting, D.R., et al., *IDF diabetes atlas: global estimates of the prevalence of diabetes for 2011 and 2030.* Diabetes Res Clin Pract, 2011. **94**(3): p. 311-21.
3. Prevention., U.S.D.o.H.a.H.S.C.f.D.C.a., *National diabetes fact sheet: national estimates and general information on diabetes and prediabetes in the United States, 2011.* 2011, Atlanta, GA: U.S. Department of Health and Human Services, Centers for Disease Control and Prevention.
4. Nichols, G.A. and J.B. Brown, *Unadjusted and adjusted prevalence of diagnosed depression in type 2 diabetes.* Diabetes Care, 2003. **26**(3): p. 744-9.
5. Mezuk, B., et al., *Depression and type 2 diabetes over the lifespan: a meta-analysis.* Diabetes Care, 2008. **31**(12): p. 2383-90.
6. Wilson, P.W., et al., *Type 2 diabetes risk in persons with dysglycemia: the Framingham Offspring Study.* Diabetes Res Clin Pract, 2011. **92**(1): p. 124-7.
7. *The link between family history and risk of type 2 diabetes is not explained by anthropometric, lifestyle or genetic risk factors: the EPIC-InterAct study.* Diabetologia, 2013. **56**(1): p. 60-9.
8. Wing, R.R., et al., *Cardiovascular effects of intensive lifestyle intervention in type 2 diabetes.* N Engl J Med, 2013. **369**(2): p. 145-54.
9. Mingrone, G., et al., *Bariatric surgery versus conventional medical therapy for type 2 diabetes.* N Engl J Med, 2012. **366**(17): p. 1577-85.
10. Avery, L., et al., *Changing physical activity behavior in type 2 diabetes: a systematic review and meta-analysis of behavioral interventions.* Diabetes Care, 2012. **35**(12): p. 2681-9.
11. Boule, N.G., et al., *Effects of exercise on glycemic control and body mass in type 2 diabetes mellitus: a meta-analysis of controlled clinical trials.* JAMA, 2001. **286**(10): p. 1218-27.
12. Schellenberg, E.S., et al., *Lifestyle interventions for patients with and at risk for type 2 diabetes: a systematic review and meta-analysis.* Ann Intern Med, 2013. **159**(8): p. 543-51.
13. Bianchi, C. and S. Del Prato, *Metabolic memory and individual treatment aims in type 2 diabetes--outcome-lessons learned from large clinical trials.* Rev Diabet Stud, 2011. **8**(3): p. 432-40.
14. Gregg, E.W., et al., *Association of an intensive lifestyle intervention with remission of type 2 diabetes.* JAMA, 2012. **308**(23): p. 2489-96.
15. Hemmingsen, B., et al., *Targeting intensive glycaemic control versus targeting conventional glycaemic control for type 2 diabetes mellitus.* Cochrane Database Syst Rev, 2013. **11**: p. CD008143.
16. Inzucchi, S.E., et al., *Management of hyperglycaemia in type 2 diabetes: a patient-centered approach. Position statement of the American Diabetes Association (ADA) and the European Association for the Study of Diabetes (EASD).* Diabetologia, 2012. **55**(6): p. 1577-96.
17. Solomon, T.P., et al., *The influence of hyperglycemia on the therapeutic effect of exercise on glycemic control in patients with type 2 diabetes mellitus.* JAMA Intern Med, 2013. **173**(19): p. 1834-6.
18. *Standards of medical care in diabetes--2011.* Diabetes Care, 2011. **34 Suppl 1**: p. S11-61.
19. Colberg, S.R., et al., *Exercise and type 2 diabetes: the American College of Sports Medicine and the American Diabetes Association: joint position statement.* Diabetes Care, 2010. **33**(12): p. e147-67.
20. Umpierre, D., et al., *Physical activity advice only or structured exercise training and association with HbA1c levels in type 2 diabetes: a systematic review and meta-analysis.* JAMA, 2011. **305**(17): p. 1790-9.
21. Umpierre, D., et al., *Volume of supervised exercise training impacts glycaemic control in patients with type 2 diabetes: a systematic review with meta-regression analysis.* Diabetologia, 2013. **56**(2): p. 242-51.
22. Boule, N.G., et al., *Meta-analysis of the effect of structured exercise training on cardiorespiratory fitness in Type 2 diabetes mellitus.* Diabetologia, 2003. **46**(8): p. 1071-81.
23. Hamilton, M.T., D.G. Hamilton, and T.W. Zderic, *Sedentary behavior as a mediator of type 2 diabetes.* Med Sport Sci, 2014. **60**: p. 11-26.

24. Wilmot, E.G., et al., *Sedentary time in adults and the association with diabetes, cardiovascular disease and death: systematic review and meta-analysis.* Diabetologia, 2012. **55**(11): p. 2895-905.
25. Reutrakul, S. and E. Van Cauter, *Interactions between sleep, circadian function, and glucose metabolism: implications for risk and severity of diabetes.* Ann N Y Acad Sci, 2014. **1311**: p. 151-73.
26. Evert, A.B., et al., *Nutrition therapy recommendations for the management of adults with diabetes.* Diabetes Care, 2013. **36**(11): p. 3821-42.
27. Sievenpiper, J.L. and P.D. Dworatzek, *Food and dietary pattern-based recommendations: an emerging approach to clinical practice guidelines for nutrition therapy in diabetes.* Can J Diabetes, 2013. **37**(1): p. 51-7.
28. Thomas, D. and E.J. Elliott, *Low glycaemic index, or low glycaemic load, diets for diabetes mellitus.* Cochrane Database Syst Rev, 2009(1): p. CD006296.
29. Thomas, D.E. and E.J. Elliott, *The use of low-glycaemic index diets in diabetes control.* Br J Nutr, 2010. **104**(6): p. 797-802.
30. Jenkins, D.J., et al., *Effect of legumes as part of a low glycemic index diet on glycemic control and cardiovascular risk factors in type 2 diabetes mellitus: a randomized controlled trial.* Arch Intern Med, 2012. **172**(21): p. 1653-60.
31. Ajala, O., P. English, and J. Pinkney, *Systematic review and meta-analysis of different dietary approaches to the management of type 2 diabetes.* Am J Clin Nutr, 2013. **97**(3): p. 505-16.
32. Ley, S.H., et al., *Prevention and management of type 2 diabetes: dietary components and nutritional strategies.* Lancet, 2014. **383**(9933): p. 1999-2007.
33. Kanfer, F.H.G.-B., Lisa, *Helping people change: A textbook of methods* ed. A.P. Frederick H.; Goldstein. Vol. Vol. 52. 1991, Elmsford, NY, US: Pergamon Press. 305-360.
34. Burke, L.E., J. Wang, and M.A. Sevick, *Self-monitoring in weight loss: a systematic review of the literature.* J Am Diet Assoc, 2011. **111**(1): p. 92-102.
35. McGrane, N., et al., *Addition of motivational interventions to exercise and traditional Physiotherapy: a review and meta-analysis.* Physiotherapy, 2014.
36. Pedersen, B.K. and B. Saltin, *Evidence for prescribing exercise as therapy in chronic disease.* Scand J Med Sci Sports, 2006. **16 Suppl 1**: p. 3-63.
37. George, D.R., L.S. Rovniak, and J.L. Kraschnewski, *Dangers and opportunities for social media in medicine.* Clin Obstet Gynecol, 2013. **56**(3): p. 453-62.
38. Drivsholm, T.B. and O. Snorgaard, *[Organization of treatment and control of type 2 diabetic patients].* Ugeskr Laeger, 2012. **174**(37): p. 2159-62.
39. Bland, J.M., *The tyranny of power: is there a better way to calculate sample size?* BMJ, 2009. **339**: p. b3985.
40. Campbell, M.J., S.A. Julious, and D.G. Altman, *Estimating sample sizes for binary, ordered categorical, and continuous outcomes in two group comparisons.* BMJ, 1995. **311**(7013): p. 1145-8.
41. Karstoft, K., et al., *The effects of free-living interval-walking training on glycemic control, body composition, and physical fitness in type 2 diabetic patients: a randomized, controlled trial.* Diabetes Care, 2013. **36**(2): p. 228-36.
42. Beck, A.T., et al., *An inventory for measuring depression.* Arch Gen Psychiatry, 1961. **4**: p. 561-71.
43. McHorney, C.A., J.E. Ware, Jr., and A.E. Raczek, *The MOS 36-Item Short-Form Health Survey (SF-36): II. Psychometric and clinical tests of validity in measuring physical and mental health constructs.* Med Care, 1993. **31**(3): p. 247-63.
44. Mortensen, E.L., et al., *Personality in late midlife: associations with demographic factors and cognitive ability.* J Aging Health, 2014. **26**(1): p. 21-36.
45. Kolin, E.A., L. Price, and I. Zoob, *Development of a Sensation-Seeking Scale.* J Consult Psychol, 1964. **28**: p. 477-82.
46. Thayer, J.F., et al., *A meta-analysis of heart rate variability and neuroimaging studies: implications for heart rate variability as a marker of stress and health.* Neurosci Biobehav Rev, 2012. **36**(2): p. 747-56.

47. Maindal, H.T., I. Sokolowski, and P. Vedsted, *Translation, adaptation and validation of the American short form Patient Activation Measure (PAM13) in a Danish version.* BMC Public Health, 2009. **9**: p. 209.
48. Gardner, B., et al., *Towards parsimony in habit measurement: testing the convergent and predictive validity of an automaticity subscale of the Self-Report Habit Index.* Int J Behav Nutr Phys Act, 2012. **9**: p. 102.
49. Schulz, K.F., D.G. Altman, and D. Moher, *CONSORT 2010 statement: updated guidelines for reporting parallel group randomised trials.* BMJ, 2010. **340**: p. c332.
50. *Uniform requirements for manuscripts submitted to biomedical journals. International Committee of Medical Journal Editors.* JAMA, 1997. **277**(11): p. 927-34.
51. Larsen, K.T., et al., *Effectiveness of a one-year multi-component day-camp intervention for overweight children: study protocol of the Odense overweight intervention study (OOIS).* BMC Public Health, 2014. **14**(1): p. 313.
52. Lund, S.S., et al., *Targeting hyperglycaemia with either metformin or repaglinide in non-obese patients with type 2 diabetes: results from a randomized crossover trial.* Diabetes Obes Metab, 2007. **9**(3): p. 394-407.
53. Ried-Larsen, M., et al., *Moderate-and-vigorous physical activity from adolescence to adulthood and subclinical atherosclerosis in adulthood: prospective observations from the European Youth Heart Study.* Br J Sports Med, 2013.
54. Ried-Larsen, M., et al., *Associations between objectively measured physical activity intensity in childhood and measures of subclinical cardiovascular disease in adolescence: prospective observations from the European Youth Heart Study.* Br J Sports Med, 2013.

# APPENDIX

EXAMPLES OF RECIPES for UTURN

UGE XX

# U TURN

2 PERSONER I 7 DAGE

Alle opskrifterne er baseret på at der spises 2/3 dele af retterne til aftensmad og at en 1/3 gemmes til frokost dagen efter.

Frokosten suppleres med gnavegrønt og grøntsagsjuice/smouthie.

DAG 1

## Torskefisk med bønner og basilikum-gremolata

25 g hasselnødder
1 potte basilikum
1 grøn citron
1 roséløg
½ salathoved
250 g grønne bønner
2 fed hvidløg
300 g flasketomater
200 g torskefisk i filet
Olivenolie, havsalt og sort peber, evt. smør

Skyl grøntsagerne.

**Gremolata**
Rist hasselnødderne på en tør pande og hak dem groft. Vend dem med basilikumblade, reven citronskal, finthakket løg og lidt olivenolie.

**Bønnesalat**
Del salaten i mindre stykker og nip bønnerne. Damp bønnerne i lidt vand med olivenolie og salt i 3-4 minutter. Lad dem afkøle let og vend dem med finthakket hvidløg, tomater i tern, lidt finthakkede stilke fra basilikummen, citronsaft, salt, peber og olivenolie. Fordel det hele ovenpå salatbladene.

**Fisk**
Gå fisken efter for ben og del den i 2-4 stykker. Steg fisken ved god varme i olivenolie, 2-3 minutter på hver side. Krydr med salt og peber.

**Servering**
Servér fisken med bønnesalaten. Top med basilikum-gremolata og lidt frisk olivenolie.

DAG 2

## Bagt og kogt hokkaido i rosmarin

1 grøn citron
½ bundt rosmarin
1 roséløg
1 hokkaido
300 g faste tomater
100 g babyleaf salat
Olivenolie, havsalt og sort peber

Skyl grøntsagerne. Tænd ovnen på 200 grader.

**Bagt hokkaido**
Skræl hokkaido-græskarret, tag kernerne ud og skær halvdelen af det i både. Vend bådene i olivenolie, salt, peber og et par rosmarinkviste. Kom bådene op i et ovnfast fad og beg dem i 20 minutter, til de er møre.

**Hokkaidopuré**
Skær resten af hokkaidoen i tern og steg dem i en gryde i olivenolie. Tilsæt lidt vand og kog igennem til de er møre. Tjek en gang imellem at gryden ikke koger tør. Purér med en gaffel eller stavblender og tilsmag med lidt citronsaft, salt og peber.

**Salat**
Skær tomaterne i tern og vend dem med salat, lidt citronskal, et finthakket løg, salt og olivenolie.

**Servering**
Servér farsbrødet i skiver med hokkaido-puré, bagt hokkaido, de friske tomater samt lidt ekstra rosmarin og citron.

DAG 3

**Braiseret kylling**
**med grønkål og æble**

200 g kyllingeoverlår
1 aubergine
½ bundt rosmarin
1 roséløg
1-2 fed hvidløg
25 g hasselnødder
150 g grønkål
½ salathoved
1 æble
Salt, peber, olivenolie og lidt eddike fx æbleeddike samt evt. smør

Skyl grøntsagerne.

**Kylling**
Krydr kyllingen med salt og peber. Skær auberginen i tern og hak løget og hvidløgsfeddene fint. Gem evt. lidt hakket løg til salaten. Brun kyllingestykkerne i en gryde sammen med aubergine, løg og hvidløg. Tilsæt 2 dl vand, lidt rosmarinkviste. Skru ned for varmen og lad kyllingen simre til den er mør. Tjek løbende at der er væde i gryden.

**Grønkålssalat**
Rist hasselnødderne på en tør pande og hak dem groft. Rib grønkålen og skær den i helt fine strimler. Vend grønkålen med salat i mindre stykker, nødder, lidt finthakket løg (kan undlades) og det saftige, nye, danske æble i tern. Tilsmag med en anelse salt, lidt eddike og olivenolie.

**Servering**
Servér kyllingen med aubergine og sky. Spis salat med grønkål, æbler og hasselnødder til.

DAG 4

## Blødkogt æg på porre med lime, stegte pebre og kapers

500 g farvede gulerødder
2 porrer
2 røde pebre
½ glas kapers i havsalt
½-1 løg
1 lime
2 æg
Olivenolie, havsalt og sort peber

Skyl grøntsagerne og skræl gulerødderne og skær dem i stave.

**Porrer**
Rens porrerne for jord. Del dem på midten og kog dem i en anelse vand med salt og olivenolie i 7-8 minutter til de er helt møre.

**Æg**
Kog æggene i 5 minutter, afkøl dem let og pil dem.

**Peberfrugter**
Skær peberfrugterne i kvarte og steg dem ved god varme i olivenolie til de er møre. De må gerne få lidt sorte mærker. Vend dem med skyllede kapers, finthakkede løg og limesaft. Hvis du gerne vil have lidt ekstra sødme i din lime, så skær den over på midten og steg den et par minutter på "kødsiden" sammen med pebrene.

**Servering**
Servér æggene på de kogte porrer sammen med de stegte pebre og de sprøde farvede gulerødder.

DAG 4

## Røget makrel med kartofler og spidskålssalat

400 g kartofler
1 lille spidskål
1 grøn citron
1 æble
1 lille røget makrel
1 rød peberfrugt
Olivenolie, havsalt og sort peber, sukker

Skyl grøntsagerne.

**Kartofler**
Skrub kartoflerne og kog dem i letsaltet vand 10-12 minutter under låg. Sluk for blusset og lad dem trække færdige. Dræn vandet fra og vend dem med lidt olivenolie.

**Spiskål**
Del kålet på midten og tag stokken ud. Kom de 2 halve spidskål op i en gyde sammen med en anelse vand, salt og olivenolie. Damp kålen i under låg i 3-4 minutter. Server den varme kål med tern af æble og citronskal.

**Makrel og peberfrugt**
Åbn makrellen og tag forsigtigt skindet af den. Pil det store ben i midten ud og pluk kødet i grove stykker. Anret det på et lille fad.
Skær peberfrugten i strimler og brug dem som snack.

**Servering**
Servér makrel med kartofler, peberfrugt og dampet spidskål.

DAG 5

# Kikærter i tikka med tomat, kartofler, grønkål og ristede cashewnødder

200 g udblødte kikærter
400 g kartofler
1 roséløg
2-3 fed hvidløg
200 g faste tomater
75 g grønkål
½ chili
1 brev tikka masala (krydderiblanding)
⅓ grøn citron
25 g cashewnødder (ristede)
Olivenolie, havsalt og sort peber

Skyl grøntsagerne og tør grønkålen godt.

**Kikærter**
Skyl kikærterne og sæt dem over at koge i friskt vand. Kog dem møre i 30-40 minutter og afdryp dem i en sigte. Gem lidt af kogevandet.

**Grønt**
Skur kartoflerne og skær dem i tern. Steg dem møre og let gyldne i olie i en jerngryde.
Pil løg og hvidløg, hak det og kom det i gryden til kartoflerne, når de er møre. Hak grønkål groft, skær tomaterne i tern og hak chilien fint.

**Kikærter i tikka**
Vend tikka masala i gryden med kartofler og løg, og rør godt rundt. Kom de møre kikærter i gryden til kartoflerne, spæd med lidt kogevand og vend tomatternene i. Kog igennem og smag til med salt, citronsaft og sort peber. Vend grønkålen i, varm kort igennem og smag til igen.

**Servering**
Servér kartofler og kikærter i tikka masala i dybe tallerkner og drys med finthakket chili og ristede cashewnødder.

# Dag 6

**Broccoli i karry**
**med 3 slags ris og tomat-topping**

200 g rismix
½ løg
300 g gulerødder
½ squash
1 peberfrugt
1 broccoli
½ chili
1 brev sumatra karry paste (krydderi-pasta)
1 dåse kokosmælk (200 ml)
100 g faste tomater
1 spsk. eddike, 1 tsk. olivenolie, havsalt og sort peber

**Ris**
Kog risene i ½ liter vand under låg ved svag varme i ca. 30 minutter. Lad dem hvile i 5 minutter og smag til med salt inden servering. Gem ca. ⅗ til dag 5.

**Grøntsager**
Skur og skyl grøntsagerne. Hak løget groft. Skær gulerødder og squash i tynde skrå skiver og peberfrugten i strimler. Gem ⅓ peberfrugt til topping. Del broccoli i små buketter og stær stokken i skiver som gulerødderne. Hak chilien fint.

**Kalkun og broccoli i karry**
Dup kalkunkødet tørt og brun det i lidt olie i en tykbundet gryde eller wok. Tilføj gulerødder, løg, squash og broccolistok og steg et par minutter. Kom sumatra karry paste ved, rør godt rundt og hæld 1 dl vand på. Lad det koge op og kom kokosmælk i. Tilføj peberfrugt og broccolibuketter og lad retten småsimre i 3-4 minutter. Spæd evt. med lidt mere vand. Smag til med lidt finthakket chili, salt og peber.

**Topping**
Skær tomater og resten af peberfrugten i små tern. Vend det sammen og smag til med chili, salt og lidt eddike.

**Servering**
Servér ris i dybe tallerkner med karryretten ovenpå og top med tomater og peberfrugt.

# Dag 7

## Quinoadeller med blomkål og karse

1 blomkål
200 g quinoa
1 bakke karse
100 g yoghurt naturel
2 æg
3 fed hvidløg
½ chili
1-2 breve hvid miso (efter smag - start med lidt)
Olivenolie, salt og peber, eddike el. citron, smør

Skyld grøntsagerne.

**Quinoa**
Kog quinoaen i 10 minutter i letsaltet vand og afkøl dem dem. Hak halvdelen af blomkålen i en hurtighakker til den ligner bulgur. Vend den med kogt quinoa og tilsæt lidt karse, æg og samt salt og peber. Lad farsen hvile i ca. 20 minutter.

**Blomkålssalat**
Skær resten af blomkålen i tynde skiver og vend dem med en dressing af misopasta rørt med presset hvidløg og 2-3 spsk. vand. Smag til med lidt chili og citron/eddike.

**Quinoadeller**
Form quinoafarsen til flade deller og steg dem gyldne og sprøde i olivenolie. Steg dem i 3-4 minutter på hver side.

**Karsecreme**
Rør yoghurten med resten af karsen og tilsmag med salt og peber.

**Servering**
Servér de gyldne quinoadeller med blomkålssalat og karsecreme.

## DAILY SELF REPORT QUESTIONNAIRES

Dette skema har to primære formål:
1) At give dig en øget bevidsthed om dine daglige valg
2) At give os mulighed for at justere eventuelle udfordringer forbundet med interventionen og støtte dig bedst muligt i den kommende proces

Derfor bedes du bruge 2-5 minutter på at udfylde nedenstående hver dag. Udfyld ved at notere et tal fra 1-10 ud for hvert område. Eksemplerne er guidelines, som skal hjælpe dig med at rangere din dag. Det er meget vigtigt, at du er ærlig også på de mindre gode dage, for det er den eneste måde, vi har en reel chance for at hjælpe dig bedst muligt.

**Dato:**

**Træning:**
1 = Jeg skulle have trænet i dag, men fik det ikke gjort
5 = Jeg trænede, men med lavere intensitet end jeg skulle
10 = Jeg gennemførte den planlagte træning eller jeg holdt hviledag, som planlagt.

*Giv din træning et tal fra 1-10:*

**Daglig fysisk aktivitet:**
1 = Jeg gik mindre end 2000 skridt i dag
5 = Jeg gik mellem 5000-6000 skridt i dag
10 = Jeg gik mere end 10.000 skridt i dag

*Giv din fysiske aktivitet et tal fra 1-10:*

**Kost:**
1 = Jeg holdt mig slet ikke til den planlagte kost i dag
5 = To ud af tre hovedmåltider gik efter planen
10 = Jeg fulgte min kostplan 100 procent

*Giv din kost et tal fra 1-10:*

**Søvn:**
1 = Jeg sov mindre end 6 timer. Min søvn var meget forstyrret og jeg havde svært ved at falde i søvn igen.
5 = Jeg sov 7-8 timer, men var vågen flere gange. Dog faldt jeg hurtigt i søvn igen.
10 = Jeg sov 7-8 timer uden større forstyrrelser.

*Giv din søvn et tal fra 1-10:*

**Stress:**
1 = Jeg har haft en meget stresset dag, som har påvirket mig uhensigtsmæssigt på andre områder
5 = Lidt stresset perioder, men også rolige perioder i løbet af dagen
10 = God stress balance og jeg har det godt

*Giv dit stressniveau et tal fra 1-10:*

**Humør og motivation**
1 = Jeg har været i dårligt humør og min motivation er lav
5 = Mit humør og min motivation kunne være bedre, men kunne også være værre
10 = Mit humør og min motivation er i top

*Giv dit humør og din motivation et tal fra 1-10:*

**Øvrige kommentarer: (Her kan du uddybe ovenstående svar eksempelvis hvis du har spist andet end den leverede mad fra Årstiderne, årsagen til manglende motivation eller hvis du bare har haft en fantastisk dag)**

## INFORMATION LETTER TO GP

Til den behandlingsansvarlige læge

Label med navn og Cpr. Nr

Ovenstående patient har accepteret at indgå i det kliniske forskningsprojekt UTURN. (videnskabsetisk forsøgsprotokol nummer: XXX)

Det drejer sig om et randomiseret klinisk forskningsprojekt omhandlende intensiv livsstilsintervention (motion, kost, søvn, coaching) af patienter med type 2 diabetes. Patienterne randomiseres til enten intensiv livsstilsintervention eller kontrolgruppe. Projektet varer 12 måneder.

I denne periode vil patientens diabetes-, hypertensions- og lipidsænkende behandling varetages af studiets behandlingsansvarlige læger og sygeplejersker med tilknytning til endokrinologisk afdeling, Rigshospitalet. Der kan i projektperioden blive tale om skift af patientens vanlige medicinske behandling.

Øjenscreening og fodkontrol skal fortsat foregå hos patientens vanlige diabetesansvarlige læge.

Patienten vil selv blive gjort opmærksom på, hvornår vanlig diabeteskontrol igen skal varetages af vanlig diabetesansvarlige læge.

Spørgsmål i forbindelse med projektet kan rettes mod:

Projektsygesplejerske XX

Centre of Inflammation and Metabolism (CIM)
Centre for Physical Activity Research (CFAS)
Rigshospitalet 7641
Blegdamsvej 9
DK-2100 Copenhagen
tel +45 3545XXX
fax +45 35457644
e-mail XXX@rh.regionh.dk

RECRUITMENT ADD

# Vi søger deltagere til med diabetes 2 til UTURN projektet.

Har du diabetes type 2 og er interesseret i at lægge din livsstil om, så er UTURN 2 måske noget for dig.

UTURN er en udløber af TV-programmet med livsstilscoachen Chris MacDonald fra 2013, hvor deltagerne forsøgte at forbedre deres sygdomsbillede kvit ved hjælp af livsstilsændringer. Vi ønsker at undersøge om erfaringerne fra TV-programmet kan overføres til en større gruppe personer med diabetes 2 uden at processen bliver belyst i et TV-program. Vi søger derfor deltagere til dette nye forskningsprojekt.

Du kan deltage i dette forsøg, hvis du:

- ✓ Har type 2 diabetes
- ✓ Ikke får anden diabetesmedicin end Metformin
- ✓ Er 18 år eller ældre
- ✓ Ikke er gravid
- ✓ Ikke lider af stofskifte-, lever-, nyre-, hjerte- eller lungesygdomme
- ✓ Ikke lider at et handicap som umuliggør, at de kan deltage i intensive motionsformer
- ✓ Har et body mass indeks højere end 25 og lavere end 40 $kg/m^2$

Projektet har til formål at undersøge om man gennem intensiv livsstilsomlægning kan forbedre blodsukkerkontrollen hos personer med diabetes type 2 til et niveau, hvor man kan mindske medicinforbruget. Projektet er omfattende og består af coaching til en personlig træning i op til seks gange pr. uge, sundere kost, og et ændret søvnmønster.

Interesserede deltagere vil blive udtrukket tilfældigt til UTURN gruppen (80 personer) eller en gruppe som ikke modtager livsstilsbehandling (40 personer).

Projektet forventes at starte i foråret 2015 og forløber over to år. I løbet af det første år skal du møde op på vores testcenter 6 gange. Her vil du få foretaget diverse undersøgelser blandt andet sukkerbelastning, test af fysisk form, blodprøvetagning, kognitive tests samt bestemmelse af muskel- og fedtmasse. Efter det andet år skal alle deltagere møde op i laboratoriet til opfølgning. Der udbetales økonomisk godtgørelse i forbindelse med testdagene.

Vil du vide mere om projektet skal du til at kontakte UTURNs projektsygeplejerske på email: xxxx@regionh.dk. Skriv lidt om din baggrund og profession, hvornår du fik type 2 diabetes, hvilke former for medicin du tager og hvor meget. Du skal ligeledes angive din kontaktinformation (Navn, adresse og telefonnummer) samt i hvilket tidsrum du kan træffes telefonisk. Så vil du blive kontaktet tidligst 2 hverdage efter vi har modtaget emailen.

Undersøgelserne foregår på Trygfonden Center for Aktiv Sundhed, Rigshospitalet, Tagensvej 20, opgang 75, 1. sal, 2100 Kbh. Ø.

**TELEPHONE SCREENING QUESTIONNAIRE**

**Telefonsamtale:**

**Personlige oplysninger:**

| | |
|---|---|
| Navn: | |
| Adresse: | |
| Telefonnummer: | |
| E-mail adresse: | |
| CPR-nummer: | |

**Inklusionskriterier:**

| | |
|---|---|
| Kendt type 2 diabetes: | Ja Nej<br>Siden: __________ (maks 5 år) |
| Sidste HbA1c ≥ 6,5% (48 mmol/mol) (langtidsblodsukker) | Ja Nej |
| Maks. 3 forskellige typer medicin mod sukkersyge | Ja Nej |
| Alder over 18 år | Ja Nej |
| BMI over 25 men under 40 kg/m$^2$ | Ja Nej |
| Accept af at medicin mod forhøjet blodsukker, forhøjet blodtryk og forhøjede kolesteroltal reguleres af UTURN's læger | Ja Nej |
| Accept af at købe og få leveret alt mad under studieperioden fra UTURN's samarbejdspartner (Aarstiderne.com) | Ja Nej |
| Accept af at købe abonnement til fitness-center anvist af UTURN. | Ja Nej |

**Eksklusionskriterier**:

| | |
|---|---|
| Sidste HbA1c > 9,0% (75 mmol/mol) | Ja Nej |

| | | |
|---|---|---|
| Nuværende insulinbehandling | Ja | Nej |
| Behandling med binyrebarkhormon indenfor de sidste 3 m neder | Ja | Nej |
| Fast nuværende behandling med non-steroide ntiinflammatoriske midler (NSAID's) | Ja | Nej |
| Behan ling mod stofskiftelidelse | Ja | Nej |
| Kontraindi ationer mod eller manglende evne til at udføre fysisk aktivitet som beskrevet i protokollen | Ja | Nej |
| Kendt ikke-behandlet blodmangel (for lav æmoglobin) | Ja | Nej |
| Kendt hjertesygdom (iskæmi k hjertesygdom, arytmi, hjertesvigt) | Ja | Nej |
| Graviditet i kl. planlagt graviditet indenfor de næste 24 måneder | Ja | Nej |

**Øvrige oplysninger:**

| | | | |
|---|---|---|---|
| Følgesygdom til sukkersyge<br>• Nervesygdom<br>• Hjerte-Karsygdom<br>• Nyresygdom<br>• Øjensygdom | Ja<br>Ja<br>Ja<br>Ja<br>Ja | Nej<br>Nej<br>Nej<br>Nej<br>Nej | Hvis ja: Beskriv sygdom og symptomer |
| Kendt leversygdom | Ja | Nej | Hvis ja: Beskriv sygdom og symptomer |
| Kendt lungesygdom | Ja | Nej | Hvis ja: Beskriv sygdom og symptomer |
| Kendt nyresygdom | Ja | Nej | Hvis ja: Beskriv sygdom og symptomer |

| | | |
|---|---|---|
| | | |
| Sygdomme i bevægeapparat | Ja Nej | Hvis ja: Beskriv sygdom og symptomer |
| Tobak | Ja Nej Tidligere<br><br>Antal pr dag aktuelt:_____________ Kumuleret antal pakkeår:____________________ | |
| Alkohol: Genstande pr. u e | | |

**Hvis 'Ja' til nogen af ovenstående: konferér med Katrine Bagge Hansen eller Kristian Karstoft om pt. kan inkluderes. Dette skal afklares før nedenstående 'Konklusion' udfyldes.**

**Konklusion:**

| | |
|---|---|
| Person indtil videre inkludérbar | Ja Nej |
| Hvis 'Nej': Må vi kontakte personen ift. fremtidige forskningsprojekter? | Ja Nej |
| Hvis 'Ja' – planlægning af forundersøgelse (screening): | Dato:__________ Klokkeslæt:___________________ |

**Oplys følgende til personen:**

- **Du skal aflevere urinprøve og have taget blodprøver på Ambulatorium for Blodprøvetagning, afsnit 5001, som ligger i stueetagen mellem opgang 4 og 5på Rigshospitalet senest 10 dage før screeningsbesøget. Blodprøvetagningen har åbent kl. 7:30 til 15:00 alle hverdage (fredag dog kun til 14:30).**
- **Du skal medbringe din fulde medicinliste til forundersøgelsen. Denne skal indeholde præparatnavn, styrke, dosering samt antal gange pr. dag medicinen indtages.**

**Telefonscreening foretaget af:_________________________________ d. ________________**

**MEDICAL SCREENING QUESTIONNAIRE**

**Blodprøve- og urinstix-gennemgang:**

**Eksklusion på baggrund af blodprøvesvar (Mette Svane der ved behov konfererer med Kathrine Bagge Hansen eller Kristian Karstoft):**

| | |
|---|---|
| HbA1c < 6,5 eller > 9,0% (48 hhv. 75 mmol/mol) | Ja Nej |
| TSH udenfor normalområde | Ja Nej |
| ALAT eller ASAT > 3 gange over øvre normalgrænse | Ja Nej |
| Hæmoglobin under nedre normalgrænse | Ja Nej |
| Kreatinin over 130 µM | Ja Nej |
| Makroalbuminuri: Albumin/kreatinin-ratio i urin > 300 mg/g | Ja Nej |

**Konklusion:**

| | |
|---|---|
| Person indtil videre inkludérbar | Ja Nej |
| Hvis 'Nej': Må vi kontakte personen ift. fremtidige forskningsprojekter? | Ja Nej |

**Vurdering foretaget af:_________________________________ d. ____________________**

**Supplerende anamnese:**

| | |
|---|---|
| Allergier overfor medicin eller plaster | Ja Nej Hvilke:___________________________ |
| Tidligere sygdomme/indlæggelser | Beskriv: |
| Hjertesygdom (udspecificer med varighed, sværhedsgrad, behandling)<br>• Iskæmisk hjertesygdom<br>• Rytmeforstyrrelser<br>• Hjertesvigt<br>• Andre hjertesy domme | Beskriv: |
| Diabetes - senkomplikationer<br>• Retinopati (øjenkontrol?)<br>• Nefropati<br>• Neuropati (fodterapeut?)<br>• Arteriel insufficiens (claudikatio, fodsår) | Beskriv: |
| Hypertension (sværhedsgrad, symptomer) | Beskriv: |
| Hyper- eller dyslipidæmi | Beskriv: |
| Øvrige organsystemer | Beskriv: |
| Medicin | Præparat, styrke, dosering, antal gange pr. dag |

| | |
|---|---|
| Accept af evt. skift af sukkersyge- blodtryks- og lipidsænkende medicin i perioden hvor UTURN projektet finder sted | Ja Nej |
| Accept af at sukkersyge- blodtryks- og lipidsænkende medicin reguleres af UTURN's endokrinologer i projektperioden | Ja Nej |
| Accept af at øvrig kontrol (inkl. øjenfoto og fodkontrol) foregår ved vanlig diabeteslæge i projektperioden | Ja Nej |

Objektiv undersøgelse:

| | | |
|---|---|---|
| Højde: ___________cm | Vægt: ____________ kg | BMI: _______________ kg/m$^2$ |
| BT – højre arm: / mmHg | BT – venstre arm: / mHg | Puls: bpm |
| St.c. | Fund: | |
| St. p | Fund: | |
| Ekstremiteter (Pulsforhold, sår, føleforstyrrelser, ødemer) | Fund: | |
| EKG | Fund: | |
| Andet relevant | Fund: | |

**Konklusion:**

| | |
|---|---|
| Person inkludérbar | Ja Nej |
| Hvis 'Nej': Eksklusionsårsag | |
| Hvis 'Nej': Må vi kontakte personen ift. fremtidige forskningsprojekter? | Ja Nej |

**Screening foretaget af:___________________________________** **d.________________**

**BUDGET**

| | 2015 | 2016 | 2017 | Total | TF (UTURN) | CFAS | TF (DocEye) |
|---|---|---|---|---|---|---|---|
| **VIP** | | | | | | | |
| **Mette Y. Johansen** | 450000 | 450000 | 450000 | 1350000 | 960000 | 0 | 390000 |
| **Chris MacDonald** | 60000 | 60000 | 60000 | 180000 | 180000 | 0 | 0 |
| | | | | | | | |
| **TAP** | | | | | | | |
| **Study Nurse 100%** | 492000 | 123000 | 0 | 615000 | 615000 | 0 | 0 |
| **Student help** | 120000 | 120000 | 120000 | 461000 | 461000 | 0 | 0 |
| **Katja Kofoed 50%** | 150000 | 150000 | 150000 | 450000 | | 450000 | 0 |
| **Louise Seier 75%** | 275000 | 275000 | 275000 | 825000 | 0 | 825000 | 0 |
| | | | | | | | |
| **Running Expenses** | | | | | | | |
| **Compensation participants** | 0 | 180000 | 90000 | 270000 | 270000 | 0 | 0 |
| **Blood sampling** | 237600 | 237600 | 237600 | 712800 | 0 | 712800 | 0 |
| **Utensilia for tests** | 59400 | 59400 | 59400 | 178200 | 0 | 178200 | 0 |
| **Publicing** | 0 | 30000 | 60000 | 90000 | 0 | 90000 | 0 |
| **Data Management** | 50000 | 50000 | | 100000 | 100000 | 0 | 0 |
| **Biostatistical assistance** | 30000 | 30000 | 30000 | 90000 | 90000 | 0 | 0 |
| **Randomization/allocation** | 20000 | 0 | 0 | 20000 | 20000 | 0 | 0 |
| **Intervention** | 1305000 | 1305000 | 0 | 0 | 0 | 0 | 2610000 |
| **Sleep testing** | 150.000 | 150.000 | | | | | 300.000 |
| | | | | | | | |
| **Equipment** | | | | | | | |
| **Polar R800** | 120000 | 0 | 0 | 120000 | 120000 | 0 | 0 |
| **Minus 80C freezer** | 100000 | 0 | 0 | 100000 | 100000 | 0 | 0 |
| | | | | | | | |
| **Total** | 3469000 | 3070000 | 1532000 | 5562000 | 2916000 | 2256000 | 3000000 |
| | | | | | | | |
| **Total budget** | | | | | | | **8472000** |

TF (UTURN): Trygfoundation grant for the UTURN research (Dkr. 2.916M)

CFAS: Expensenses covered by CFAS (Dkr. 2.256M)

TF (DocEye): Trygfoundation grant for the implementation of the intervention (Dkr. 3M)
